# Local endoreduplication of the host is a conserved process during Phytomyxea–host interaction

**DOI:** 10.3389/fmicb.2024.1494905

**Published:** 2025-02-05

**Authors:** Michaela Hittorf, Andrea Garvetto, Marianne Magauer, Martin Kirchmair, Willibald Salvenmoser, Pedro Murúa, Sigrid Neuhauser

**Affiliations:** ^1^Department of Microbiology, Universität Innsbruck, Innsbruck, Austria; ^2^Department of Botany, Universität Innsbruck, Innsbruck, Austria; ^3^Department of Zoology, Universität Innsbruck, Innsbruck, Austria; ^4^Laboratorio de Macroalgas y Ficopatología, Instituto de Acuicultura, Universidad Austral de Chile, Puerto Montt, Chile

**Keywords:** endocycle, *Plasmodiophora brassicae*, *Maullinia ectocarpii*, biotroph, plant pathogen

## Abstract

**Background:**

Endoreduplication, a modified cell cycle, involves cells duplicating DNA without undergoing mitosis. This phenomenon is frequently observed in plants, algae, and animals. Biotrophic pathogens have been demonstrated to induce endoreduplication in plants to secure more space or nutrients.

**Methods:**

In this study, we investigated the endoreduplication process triggered by two phylogenetically distant Rhizaria organisms—*Maullinia spp*. (in brown algae) and *Plasmodiophora brassicae* (in plants)—by combining fluorescent *in situ* hybridization (FISH) with nuclear area measurements.

**Results:**

We could confirm that *Plasmodiophora brassicae* (Plasmodiophorida) triggers endoreduplication in infected plants. For the first time, we also demonstrated pathogen-induced endoreduplication in brown algae infected with *Maullinia ectocarpii* and *Maullinia braseltonii* (Phagomyxida). We identified molecular signatures of endoreduplication in RNA-seq datasets of *P. brassicae*-infected *Brassica oleracea* and *M. ectocarpii*-infected *Ectocarpus siliculosus*.

**Discussion:**

Cell cycle switch proteins such as CCS52A1 and B in plants, CCS52 in algae, and the protein kinase WEE1 in plants were upregulated in RNA-seq datasets hinting at a potential role in the phytomyxean-induced transition from mitotic cell cycle to endocycle. By demonstrating the consistent induction of endoreduplication in hosts during phytomyxid infections, our study expands our understanding of Phytomyxea–host interaction. The induction of this cellular mechanism by phytomyxid parasites in phylogenetically distant hosts further emphasizes the importance of endoreduplication in these biotrophic interactions.

## 1 Introduction

Endoreduplication is a process where the nuclear DNA is multiplied without subsequent cell division (Barlow, 1978; Joubès and Chevalier, 2000), resulting in endopolyploidy where the chromosome number of cells and the cell size increase (Joubès and Chevalier, 2000). Endoreduplication has been found in yeasts (Harari et al., 2018), invertebrates (Flemming et al., 2000; Smith and Orr-Weaver, 1991), mammals (Gandarillas et al., 2018), as well as in green (Horinouchi et al., 2019) and brown algae (Bothwell J. H. et al., 2010; Garbary and Clarke, 2002). The reason why many organisms maintain such an alternative cell cycle is still not well understood (De Veylder et al., 2011), but endoreduplication has often been discussed as a cellular response to mitigate stress by increasing cell size, gene copy number, and levels of gene expression (Paige, 2018; Van de Peer et al., 2021). Endoreduplication is especially prevalent in higher plants, where it plays a vital role in growth and development (Joubès and Chevalier, 2000; Lee et al., 2009). In plants, endoreduplication is usually observed when cells shift from cell proliferation and growth to cell differentiation (Joubès and Chevalier, 2000) and is often associated with an increase in cell size and cell expansion (Chevalier et al., 2011; Wildermuth et al., 2017). Gene expression drastically changes in cells undergoing endoreduplication (Bourdon et al., 2012) as seen in tomato fruits, where endoreduplication is hypothesized to increase the metabolic capacity of the plant and promote growth (Bourdon et al., 2012; Lee et al., 2009). Endoreduplication was found in brown algae; however, detailed studies on its role and regulation have not been conducted to date (Bothwell J. H. et al., 2010; Garbary and Clarke, 2002). Endoreduplication was found in the genome of the model brown alga *Ectocarpus siliculosus*, along with some general cell cycle-related genes, including cyclins, cyclin-dependent kinases, the Wee1 kinase, and a cell cycle switch protein (CCS52) homolog (Bothwell J. H. et al., 2010; Bothwell J. H. F. et al., 2010).

In contrast, the regulation of endoreduplication in plants has been well-studied. The cell cycle is controlled by oscillating cyclin-dependent kinases (CDKs) and their interaction with cyclins (CYCs) (De Veylder et al., 2003; Inzé and De Veylder, 2006). Each cell cycle phase (S, M, G1, and G2) is regulated by its specific set of cyclins and CDKs. For a review of the cell cycle and its detailed regulation in plants see Qi and Zhang (2020) and Shimotohno et al. (2021). For the transition from the regular cell cycle to the endocycle, mitosis-specific cyclins and CDKs need to be inactivated (Bhosale et al., 2019). This transition can be regulated/activated through different pathways, mainly through CDK inhibitors, selective degradation of cyclins via cell cycle switch protein-mediated activation of the anaphase-promoting complex (APC), and potential post-translational modifications (including the WEE1 kinase) of CDKs (Tourdot et al., 2023). The regulation of the endocycle is slightly different depending on the tissue. In *Arabidopsis thaliana* roots, the endocycle onset is marked by inactivation of the cyclin CYCA2;3 which is controlled through the cell cycle switch protein CCS52A1, an activator of the anaphase-promoting complex (Boudolf et al., 2009). For a detailed review of the control and development of endoreduplication in plants see De Veylder et al. (2011) and Lang and Schnittger (2020).

The obligate biotrophic plant parasitic protist *Plasmodiophora brassicae* induces endoreduplication in infected cells of *A. thaliana* (Olszak et al., 2019). *P. brassicae* belongs to the Phytomyxea (Rhizaria), which are parasites of plants, brown algae, diatoms, and oomycetes (Burki et al., 2010; Neuhauser et al., 2011). The Phytomyxea are divided into the terrestrial Plasmodiophorida, the marine Phagomyxida, and the marine Marinomyxa clade (Hittorf et al., 2020; Kolátková et al., 2021). Phytomyxea have a complex life cycle with two phases of infection: the short-lived primary (sporangial) infection and the secondary (sporogenic) infection, which often leads to hypertrophy and gall formation in their hosts (Olszak et al., 2019). During the early phase of secondary infection, the parasite keeps the infected cells in the mitotic cell cycle to promote proliferation (Devos et al., 2006; Malinowski et al., 2019) and carbohydrates are redirected toward the parasite-infected cells (Walerowski et al., 2018). While the secondary plasmodia of *P. brassicae* grow, the host cells change from cell proliferation to cell enlargement and from the mitotic cell cycle to the endocycle (Liu et al., 2020; Olszak et al., 2019). This results in local clusters of hypertrophied cells and *P. brassicae* which are so abundant and large that they lead to the formation of galls in the roots of the host plant (Malinowski et al., 2019). Long-lived resting spores are the final stage of development. These resting spores are eventually released into the soil when the infected roots degrade (Kageyama and Asano, 2009), remaining dormant until conditions are favorable for zoospore germination (Kageyama and Asano, 2009; Wang et al., 2022).

*Maullinia ectocarpii* is an example of a phytomyxean parasite that infects brown algae (Maier et al., 2000). Brown algae are photosynthetic organisms and primary producers in marine environments, yet they are taxonomically unrelated to angiosperms. This makes comparative studies with these organisms compelling from both evolutionary and biological points of view. Thus far, no spore formation of *M. ectocarpii* has been documented microscopically, although there is persuasive evidence for the presence of the secondary, gall-inducing stage on kelp sporophytes (Mabey et al., 2021). Another clue that *M. ectocarpii* can fulfill a full life cycle is the presence of resting spores in the closely related *Maullinia braseltonii*, which commonly infects the bull kelp *Durvillaea* spp. (Murúa et al., 2017). The sporangial phase of the life cycle of *M. ectocarpii* has the advantage that it can be cultured in the laboratory on suitable brown algal hosts and it is, therefore, available for experimentation (Maier et al., 2000). Brown algae infected with *M. ectocarpii* show hypertrophied-infected cells, and the host nuclei appear enlarged (Maier et al., 2000), similar to what was found in *P. brassicae*-infected plant cells. The biological relevance and the mechanisms that lead to enlarged nuclei and hypertrophy in the host cell are unknown.

Endoreduplication is important during the establishment and maintenance of biotrophic interactions in plants. Many plant biotrophs induce endoreduplication during host colonization, including mutualists such as arbuscular mycorrhizal fungi (AMF) and rhizobia, as well as parasites such as root-knot and root-cyst nematodes or powdery mildew fungi (Carotenuto et al., 2019; De Almeida Engler et al., 2012; Fan et al., 2022; Wildermuth et al., 2017). Until now, endocycles induced by symbionts (parasites and mutualists) have just been recorded in plants and are unknown in the interaction between biotrophs and marine brown algae. Endoreduplication and the reprogramming of the host cell cycle have been studied in *A. thaliana* infected with *P. brassicae* (Malinowski et al., 2019; Olszak et al., 2019), while anecdotal evidence in older studies provides information about enlarged host nuclei during infections with the phytomyxids *M. ectocarpii* and *Sorosphaerula veronicae* (Blomfield and Schwartz, 1910; Maier et al., 2000).

This study aims to establish whether induction of endoreduplication in infected host cells is a shared, evolutionary conserved mechanism in the class Phytomyxea, used to create space and obtain nutrients and energy from their hosts. To study this, we established a comparative approach, which allowed us to test whether local endoreduplication was induced not only by *P. brassicae* in plant hosts but also during the colonization of brown algae with *Maullinia spp*. We used a combination of microscopy, ploidy measurements, and molecular datasets (RNA-seq) from *P. brassicae*-infecting *Brassica spp*. and *M. ectocarpii*-infecting *E. siliculosus*, along with microscopy observations of *M*. *braseltonii*-infecting *D. incurvata*, to analyze the induction of endoreduplication. Based on these findings, we provide synergistic evidence supporting the important role of endoreduplication in phytomyxean growth, its potential involvement in local energy sink induction, and the identification of new developmental features in plant and brown algal hosts.

## 2 Materials and methods

### 2.1 Sampling, plant material, algae material, and growth conditions

#### 2.1.1 Field sampling of infected and uninfected plant material

*Plasmodiophora brassicae*-infected material of *Brassica rapa subsp. pekinensis* (root galls) was collected in a commercial field in Völs, Tyrol on 17 and 28 September 2021. Roots from healthy, uninfected control plants (*B. rapa subsp. pekinensis)* were harvested in a field in Innsbruck, Tyrol, on 30 September and 4 October 2021. Root galls from the infected plants and roots from the healthy plants were rinsed with tap water and stored at 4°C until further use as described below.

*Maullinia braseltonii*-infected material of *Durvillaea incurvata* (characterized by yellow galls) and healthy *D. incurvata* were sampled at the coast of Estaquilla, Chile on 19 May 2022. Samples (blades from infected and from healthy *Durvillaea*) were cut into 2 cm pieces and fixed with 4% Histofix (phosphate-buffered formaldehyde solution, Carl Roth). The samples were stored at 4°C until further use.

#### 2.1.2 Maintenance of *Maullinia ectocarpii*-infected *Ectocarpus siliculosus* Ec32m and *Maullinia ectocarpii*-uninfected *E. siliculosus* Ec32m cultures

*Maullinia ectocarpii* (CCAP 1538/1) was grown in *Ectocarpus siliculosus* Ec32m (CCAP 1310/4). Healthy, uninfected *E. siliculosus* Ec32m (CCAP 1310/4) was used as a control and grown in the same conditions. Cultures were maintained in artificial seawater with half-strength-modified Provasoli (West and McBride, 1999) at 15°C with a 12-h photoperiod, 20 micromol photon m^−2^s^−1^ as described in Badstöber et al. (2020a). Cultures were regularly checked for infections. The cultures were harvested and used in the experiments as described below. An overview of the infected material is shown in Supplementary Figure 6.

### 2.2 Preparation of material for microscopy and nuclear measurements

#### 2.2.1 Fixation

The samples were fixated as described in Garvetto et al. (2023). Plant roots (root galls of *Brassica rapa subsp. pekinensis* infected with *Plasmodiophora brassicae* and roots of control-uninfected *B. rapa subsp. pekinensis* plants) were cut with a razor blade and fixed with 4% Histofix (phosphate-buffered formaldehyde solution, Carl Roth) for ~1 h. Afterward, the samples were rehydrated in a series of ethanol washing (10 min 50% EtOH, 2 × 10 min 70% EtOH, final storage in 100% EtOH at −20°C). Algal samples (*Maullinia ectocarpii*-infected *Ectocarpus siliculosus* Ec32m cultures and *Maullinia ectocarpii-*uninfected control *E. siliculosus* Ec32m cultures) were fixed the same way except for an additional 2.5 min 30% H_2_O_2_ incubation step after fixation in 4% Histofix to make the cell wall more permeable for the FISH probe.

#### 2.2.2 Fluorescence In Situ Hybridization (FISH) and Hoechst staining

To prevent photobleaching, the steps were performed under red light. Algal samples (infected and healthy *E. siliculosus*) and plant samples (infected and healthy *B. rapa*) were treated in the same way. FISH was performed as described in Schwelm et al. (2016). Fixed samples were incubated for 10 min in 35% hybridization buffer (900 mM NaCl, 20 mM Tris–HCl, 35% formamide, 0.01% SDS). The hybridization buffer was removed and 100 μl of hybridization buffer—probe [Supplementary Table 7; Pl_LSU_2313 for *P. brassicae* (Schwelm et al., 2016) and MauJ17 for *M. ectocarpii*] mix (90 μl of hybridization buffer and 10 μl of probe)—was added. The sample was incubated at 46°C overnight. The samples were washed twice with 35% washing buffer (900 mM NaCl, 20 mM Tris–HCl, 5mM EDTA, and 0.01% SDS) for 20 min at 48°C. For the nuclei staining, the samples were additionally incubated in Hoechst 33342 (Thermo Fisher Scientific, Germany) for 10 min and mounted in VECTASHIELD (H-1000, Vector Laboratories). Each slide was covered with a coverslip and sealed with nail polish. The slides were stored at −20°C in darkness or immediately used.

Fixed samples of infected and healthy *D. incurvata* were cut into thin sections with a scalpel and stained with Hoechst 33342 (Thermo Fisher Scientific, Germany) for 20 min and mounted in VECTASHIELD (H-1000, Vector Laboratories). Each slide was covered with a coverslip and sealed with nail polish. The slides were stored at −20°C in darkness or immediately used.

### 2.3 Microscopy

Fluorescence microscopy was performed as described in Garvetto et al. (2023). In brief, the samples were observed using a Nikon Eclipse Ti2-E (Nikon, Japan) microscope equipped with an Andor Zyla 5.5sCMOS monochrome camera (Andor Technology, United Kingdom) using Nikon CFI Plan-Fluor 40 × /0.75 NA and 60 × /0.85 NA objectives. The excitation wavelengths for Hoechst 33342 and FISH probes were 365 and 490 nm, respectively. Negative controls without the probe but with hybridization buffer and Hoechst 33342 were included. Overlays of the different channels (DIC, channel for Hoechst, and channel for the FISH probe) and measurements were conducted using the NIS Elements software AR 5.21.03 (Nikon, Japan).

### 2.4 Transmission electron microscopy (TEM)

The root galls of *Brassica rapa subsp. pekinensis* and the roots of healthy control plants were rinsed with tap water. The samples were preselected and screened under the microscope for infections. Transmission electron microscopy was performed as described in Garvetto et al. (2023). Selected samples were chemically fixed with 2.5% glutaraldehyde in 0.1 M cacodylate buffer containing 10% sucrose at 4°C for 1 h. They were washed with cacodylate buffer and post-fixed with 1% osmium tetroxide in 0.05 M cacodylate buffer for 1 h at 4°C. This was followed by another washing with cacodylate buffer. After dehydration with an increasing acetone series, the samples were embedded in Embed 812 resin. A diamond knife (Diatome, Switzerland) and an Ultracut UCT (Leica, Austria) were used to cut cross-sections of uninfected control roots and infected root galls. The samples were mounted on grids and stained with lead citrate. A Libra 120 energy filter transmission electron microscope (Zeiss, Germany) equipped with a TRS 2 × 2k high-speed camera (Tröndle, Germany) and ImageSP software (Tröndle, Germany) was used for imaging.

### 2.5 Flow cytometry

Flow cytometry was performed as described in Suda et al. (2007) with some modifications explained in detail in the Supplementary Methods. The used standards (*Bellis perennis* for *Brassica rapa subsp. pekinensis* and *Solanum pseudocapsicum* for *Ectocarpus siliculosus*) were used because of their similar but not overlapping genome size with the used material.

### 2.6 Identification of cell cycle-related genes in infected hosts

Three publicly available RNA-seq datasets were analyzed to examine the cell cycle-related genes in phytomyxid-infected hosts. The first was from *Brassica oleracea subsp. gongylodes* infected with *Plasmodiophora brassicae* [(Ciaghi et al., 2019); BioProject: PRJEB26435], the second from *Brassica rapa subsp. pekinensis* [(Jia et al., 2017); BioProject: PRJNA322393], and the third from *Ectocarpus siliculosus* Ec32m (strain CCAP 1310/4) infected with *Maullinia ectocarpii* [strain CCAP 1538/1; (Garvetto et al., 2023); BioProject: PRJNA878940]. The inferred proteomes were searched for cell cycle-related genes using identity thresholds of above 50% of peptides and above 80% for transcripts. Keyword searches based on gene models from *E. siliculosus* to *B. oleracea* and vice versa were used to identify additional potential cell cycle-related homologous. Additionally, log_2_fold change values were extracted (Supplementary Tables 2–4). The most important genes involved in endoreduplication are summarized in Supplementary Table 1 and compared to the literature. A more detailed description of the pipeline can be found in the Supplementary Methods.

### 2.7 Statistical analysis

R studio vs. R.4.3.1 was used to analyze the nuclear measurement data. The Brown–Mood median test (accounting for data not normally distributed and unequal variances, from package “coin”) was used as a non-parametric alternative to the Student's *t*-test to assess whether the differences between the medians were significant. Additionally, a Wilcox–Mann–Whitney test (“rstatix” package) was used to assess whether the distributions of the nuclear areas were significantly different. Violin plots were used to display the differences between the distributions of the nuclear areas of infected and uninfected host cells using the package “ggstatsplots” (Patil, 2021).

## 3 Results

### 3.1 The shape and size of host nuclei depend on the developmental status of the colonizing Phytomyxea

**Figure 1:**
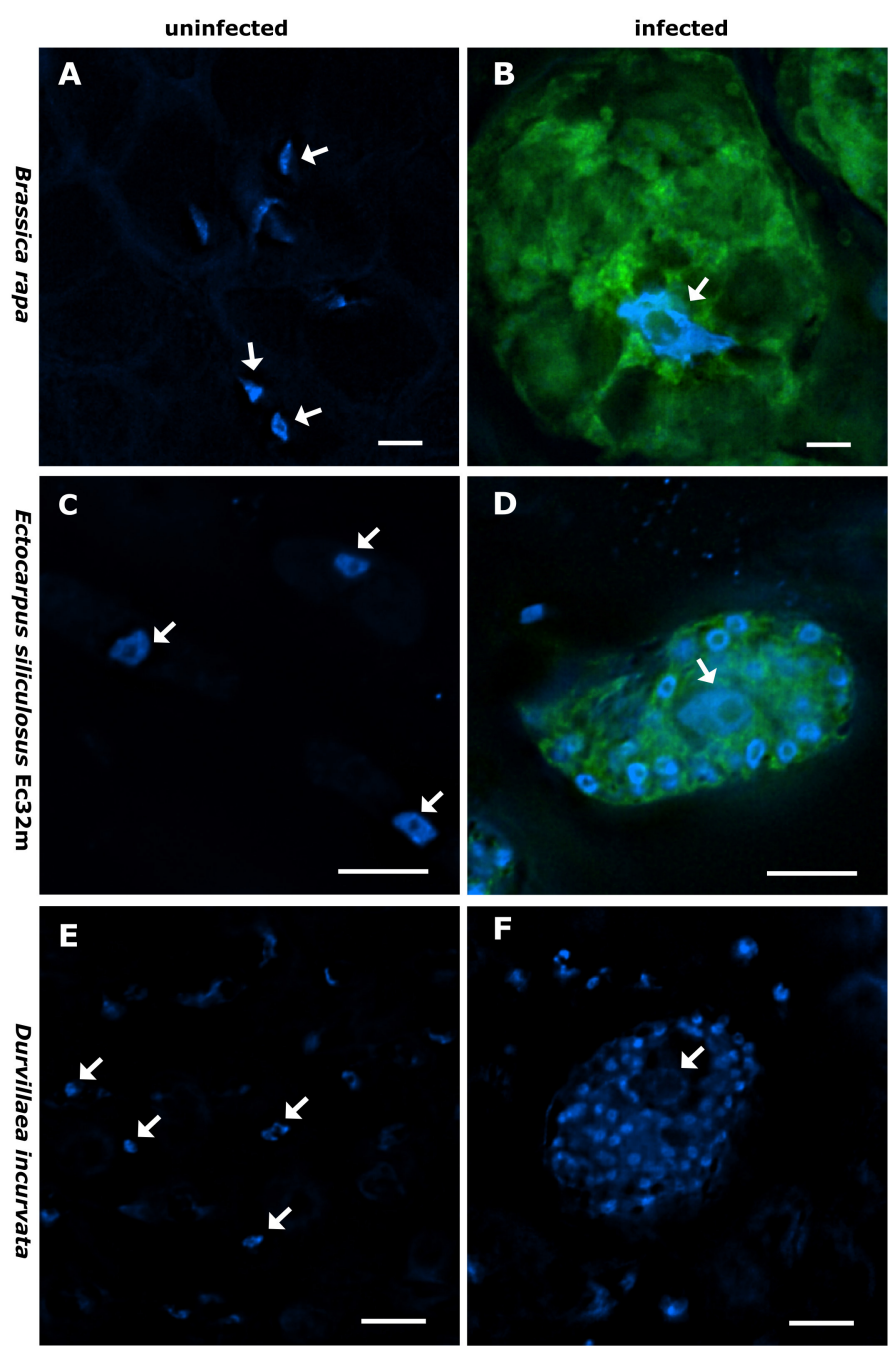
Nucleus size and shape vary between infected and non-infected hosts. *Brassica rapa* subsp. *pekinensis-*uninfected control plant **(A)**, plasmodium of *Plasmodiophora brassicae* in *B. rapa* subsp. *pekinensis* **(B)**, *Ectocarpus siliculosus* Ec32m-uninfected control culture **(C)**, multinucleate plasmodium of *Maullinia ectocarpii* in *E. siliculosus* Ec32m **(D)**, uninfected control *Durvillaea incurvata* **(E)**, and multinucleate plasmodium of *Maullinia braseltonii* in *D. incurvata* **(F)**. Overlay of Hoechst [blue signal, note the smaller nuclei of the phytomyxean plasmodium surrounding the bigger nucleus of the host (arrow)] and FISH (green signal) staining of Phytomyxea **(A–D)**; Hoechst staining only **(E, F)**. Arrows point toward host nuclei. Scale bar: 10 μm.

**Figure 2:**
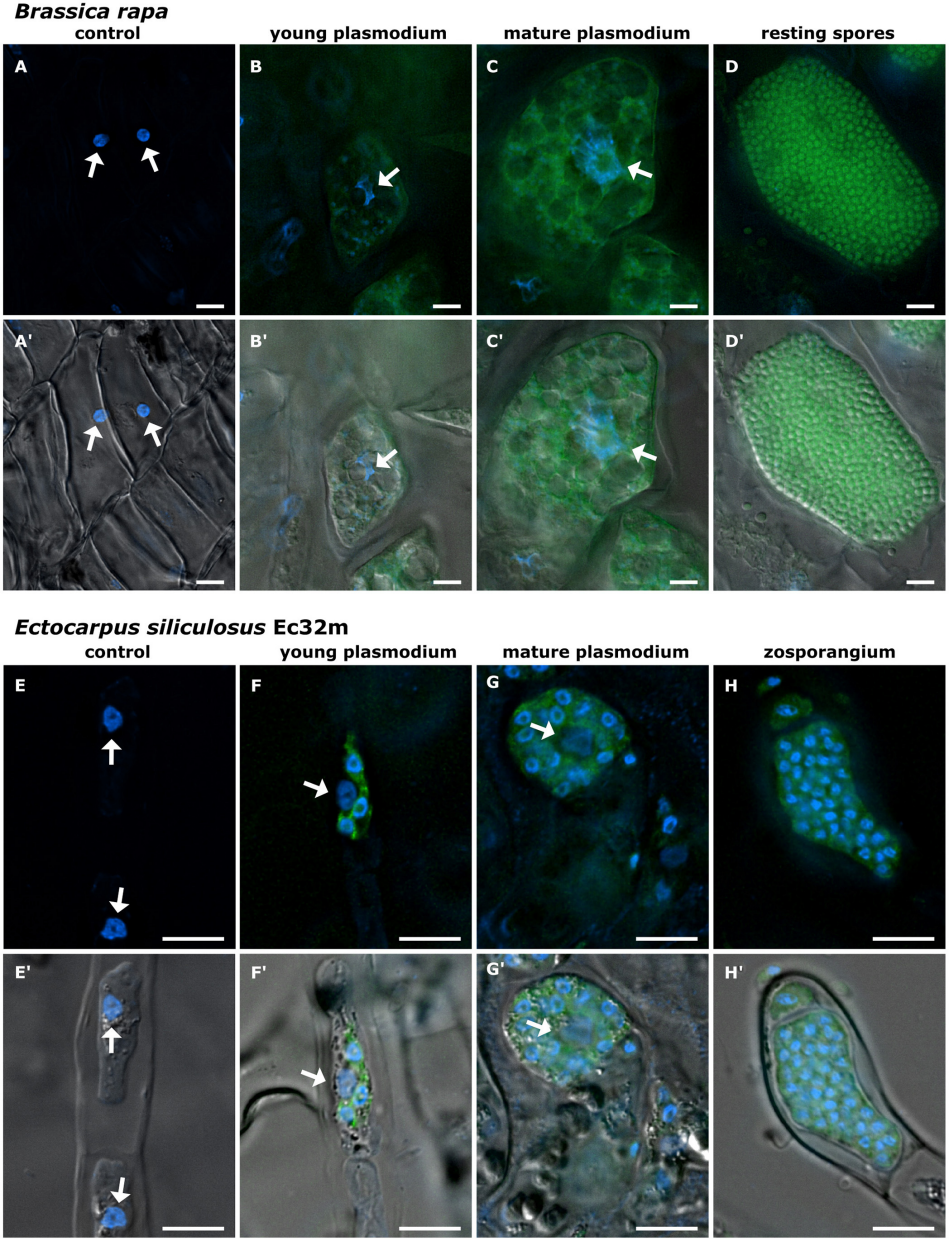
Development of Phytomyxea and the enlargement of host nuclei progress in parallel. *Brassica rapa* cells colonized by *Plasmodiophora brassicae* **(A–D, A′-D′)** and *Ectocarpus siliculosus* colonized by *Maullinia ectocarpii* **(E–H, E′-H′)**. Uninfected cells of healthy *B. rapa* control plants **(A, A′)**, young secondary plasmodium of *P. brassicae* (green) in an enlarged colonized host cell, host nucleus (arrow) already enlarged **(B, B′)**. Mature secondary plasmodium of *P. brassicae* occupies the now hypertrophied cell and engulfs the enlarged host nucleus (arrow) **(C, C′)**. *E. siliculosus* cells from healthy control cultures **(E, E′)** in comparison to colonized host cells with different infection stages of *M. ectocarpii* **(F–H; F′-H′)**. Recent infection of *M. ectocarpii* (green) in *E. siliculosus*, no hypertrophy visible yet **(F, F′)**. Mature plasmodium of *M. ectocarpii*, colonized host cell is hypertrophied and the host nucleus is enlarged (arrow) **(G, G′)**. Zoosporangium with zoospores of *M. ectocarpii* occupying the hypertrophied cell of *E. siliculosus*, no host nucleus visible **(H, H′)**. **(A–H)** Overlay of Hoechst (blue signal, visualized at 365 nm) and FAM (490 nm); **(A′-H′)** overlay of Hoechst (365 nm), FAM (490 nm), and DIC. *P. brassicae* and *M. ectocarpii* are visualized in green with FISH. Arrows point toward the host nuclei. Scale bars: 10 μm.

**Figure 3:**
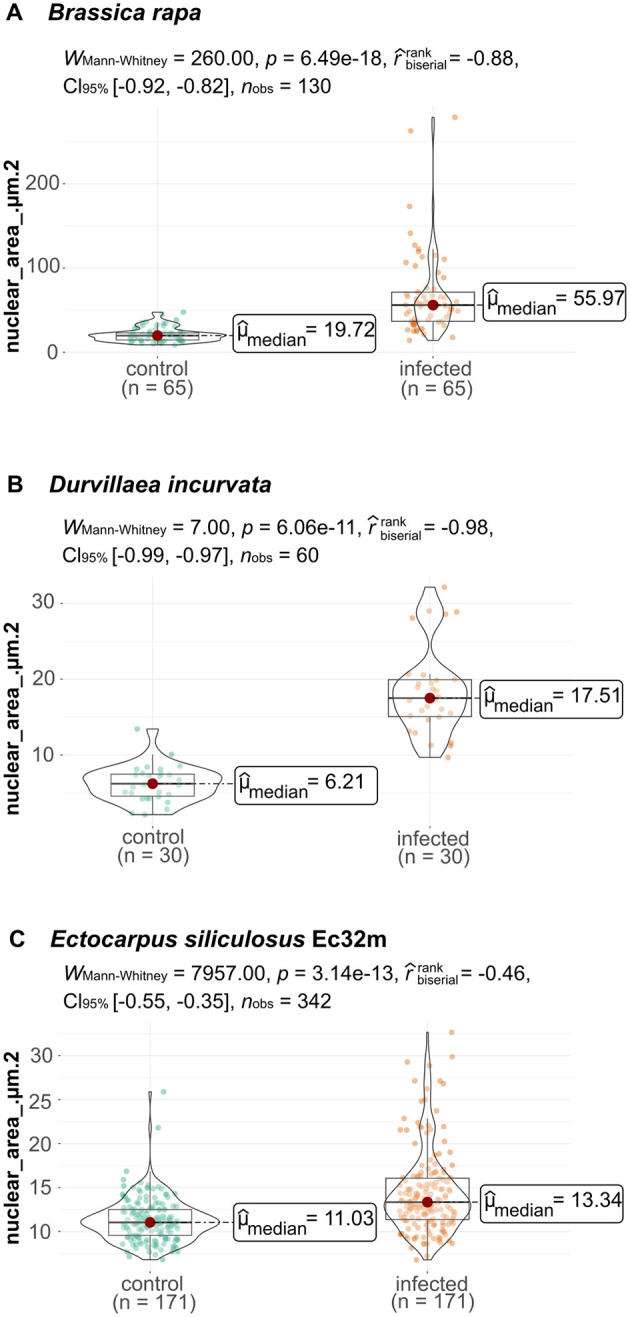
Nuclear area of phytomyxean-infected hosts differs from that of non-infected hosts. **(A)** Size of the nuclear areas of 65 *P. brassicae* colonized *B. rapa* cells (infected plant) and 65 *B. rapa* cells (healthy control plant). **(B)** Size distribution of 30 cells from healthy control *D. incurvata* and 30 cells from *M. braseltonii* colonized *D. incurvata* cells. **(C)** Size of the nuclear area of 171 cells from healthy control *E. siliculosus* Ec32m cultures compared to the nuclear area of 171 *M. ectocarpii* colonized *E. siliculosus* Ec32m cells. The distribution of nuclear areas from infected material differs significantly (Wilcox–Mann–Whitney test) from the distribution of nuclear areas from uninfected material in all comparisons.

The nuclei of *Brassica rapa subsp. pekinensis* roots from healthy plants, not infected with *P. brassicae*, were oval to round in shape and showed little variation in overall size and shape (Figures 1A, 2A, A′, Supplementary Figure 1A). The median nuclear area of uninfected plant cells was 19.72 μm^2^ [standard deviation (SD) = 8.5, *n* = 65] with a minimum of 8.76 μm^2^ and a maximum nuclear area of 47.54 μm^2^ (Figure 3A, Supplementary Figure 5). The median nuclear area of *B. rapa* cells colonized by *P. brassicae* was 55.97 μm^2^ (SD = 48.9, *n* = 65), with a minimum of 14.03 μm^2^ and a maximum of 278.93 μm^2^ (Figure 3A). The difference between the nuclear area of cells from infected and uninfected plants was highly significant (based on the Brown–Mood median test, *p* = 2.2e-16), and the nuclear area of cells from *P. brassicae*-infected plants was 2.8 times bigger based on the median size. While the nuclei of non-colonized root cells in healthy plants were similar in size and round to oval in shape, the nuclei of cells colonized by *P. brassicae* from infected plants varied in size and had a convex, bulged appearance (Figures 1A, B, 2A–D, A′-D′, Supplementary Figures 1A, B). During the colonization of cortical cells of the plant, the plasmodium of *P. brassicae* was gradually growing and occupying more and more space within the host cell. The colonized host cells were increasingly hypertrophied, and their nuclei became larger over time (Figures 2B, B′, C, C′). When the colonized cell was completely filled with resting spores of the parasite (Figures 2D, D′), the host nucleus disappeared.

Nuclei in healthy *E. siliculosus* (Ec32m) cultures had a uniform size and shape (Figures 1C, 2E, E′, Supplementary Figure 1C). The median nuclear area of *E. siliculosus* cells was 11.03 μm^2^ (sd=2.5, n=171) with a minimum of 6.8 μm^2^ and a maximum of 25.88 μm^2^ (Figure 3B, Supplementary Figure 5). The median nuclear area of *E. siliculosus* cells colonized by *M. ectocarpii* was 13.34 μm^2^ (SD = 4.8, *n* = 171) with a minimum of 6.78 μm^2^ and a maximum of 32.65 μm^2^ (Figure 3B). Based on the median sizes, the nuclei of colonized cells were 1.2 times bigger than in unaffected cells, and the difference was highly significant (based on the Brown–Mood median test, *p* = 2.15e-11). Unaffected brown algae cells had spherical to ellipsoidal nuclei with little variation in size, while nuclei of colonized cells showed a high variation in size and a higher variation in shape (Figures 1C, D, 2E–H, E′-H′, Supplementary Figures 1C, D). The nuclei of infected brown algae gradually increased in size as the parasite plasmodium developed. Cells colonized by a young plasmodium retained a normal-sized nucleus (Figures 2F, F′), while cells with a mature plasmodium exhibited enlarged nuclei (Figures 2G, G′). When the plasmodium differentiated into zoospores and filled the entire host cell, the host nucleus began to disappear (Figures 2H, H′).

We observed both, plasmodia and resting spores of *Maullinia braseltonii* in the tissue between the cortex and the medulla of infected *Durvillaea* blades. The median nuclear area of healthy *Durvillaea incurvata* cells was 6.21 μm^2^ (SD = 2.4, *n* = 30) with a minimum of 2.12 μm^2^ and a maximum of 13.43 μm^2^ (Figure 3C, Supplementary Figure 5). The nuclei of *D. incurvata* cells colonized by *M. braseltonii* appeared enlarged (Figure 1F, Supplementary Figure 1F′). The median nuclear area of infected *Durvillaea* cells was 17.51 μm^2^ (SD = 5.8, *n* = 30) with a minimum of 9.68 μm^2^ and a maximum of 32.15 μm^2^ (Figure 3C). The difference between host nuclei in colonized and unaffected cells was highly significant (based on the Brown–Mood median test, *p* = 7.551e-13). The nuclei of colonized cells were 2.8 times bigger than the nuclei of unaffected cells (based on the median of the nuclear area).

The TEM images confirmed that the host nuclei of infected *B. rapa* were not apoptotic, but that the increase in size was because of endoreduplication. The TEM images showed that the host nucleus of the colonized *B. rapa* cell had an intact membrane without holes as would be expected in case of apoptosis. Nucleoli and heterochromatin were present, indicating an active host nucleus (Supplementary Figures 2, 3).

Flow cytometry analysis of infected plant and algal material (together with healthy controls) supported the findings of the nuclear measurements and microscopy. *B. rapa* infected with *P. brassicae* showed ploidy levels of 8C and sometimes a few 16C nuclei were detected (Supplementary Figure 4B), while the roots of control plants had ploidy levels of 4C and rarely 8C (Supplementary Figure 4A). In the healthy *E. siliculosus* samples, only one ploidy level was detected (Supplementary Figure 4C). In the infected *E. siliculosus* samples, different ploidy levels could be detected; however, the interpretation of the peaks should be approached with caution, as the peaks are often not well separated and ambiguous (Supplementary Figure 4D).

### 3.2 Phytomyxea induce endocycle-related transcriptional changes

By querying available RNA-seq datasets, we could find genetic signatures pointing toward the induction of endocycle-related processes in *Brassica oleracea subsp. gongylodes* and *B. rapa subsp. pekinensis* infected with *P. brassicae* (data from Ciaghi et al., 2019; Jia et al., 2017). In those datasets, the transcripts linked to the switch from the mitotic cell cycle to the endocycle (as described for *A. thaliana* by Olszak et al., 2019; Supplementary Tables 1–3). The changes involved the upregulation of the cell cycle switch protein CCS52A1 and the upregulation of genes for the progression from G1 to S phase. In contrast, no definitive pattern was identified for the G2/M transition (Supplementary Tables 1–3).

The genetic and molecular mechanisms of the endocycle in brown algae are not fully known, but they are thought to be conserved in eukaryotes (Bothwell J. H. et al., 2010). Therefore, we analyzed the publicly available RNA-seq dataset of *E. siliculosus* infected with *M. ectocarpii* (Garvetto et al., 2023). Note that the term Ectsi refers to transcripts of *Ectocarpus siliculosus*. Transcripts of Ectsi FZR1 (or CDH1/CCS52), a homolog to the positive endocycle regulator CCS52A in plants, were upregulated in infected *E. siliculosus* (Ec32m) (Supplementary Table 1). Wee1 (the plant homolog WEE1 is important for endocycle onset in specific tissues in plants) was downregulated in infected algae (Supplementary Table 1). Transcripts of the anaphase-promoting complex/cyclosome (APC/C), which is activated through CCS52A, were both up and downregulated (Supplementary Table 4). The expression of genes responsible for regulating the G2-M transition during mitosis was found to be downregulated in infected algae when compared to uninfected hosts. Ectsi CDKA2/CDKB, whose diatom homolog is important for the G2-M transition, was downregulated in infected brown algae (Supplementary Table 1). Both A-type cyclins and B-type cyclins were downregulated in infected algae (Supplementary Table 4). CDKA1, important for the G1/S transition (i.e., DNA replication), was upregulated in *M. ectocarpii*-infected *E. siliculosus* (Supplementary Table 1). There was an upregulation of certain transcripts of D-type cyclins, whose plant homologs are involved in the G1/S transition, while others were downregulated. Additionally, E2F, a positive transcriptional regulator of the G1/S transition, was upregulated in infected algae, while RBR, its inactivator, was downregulated (Supplementary Tables 1, 4).

The predicted proteomes of *M. ectocarpii* and *P. brassicae* were filtered for putative effectors and those were further filtered by the COG category “cell cycle” (D), to identify putative effectors interacting with the cell cycle regulation of their host to actively induce endoreduplication (Supplementary Tables 5, 6). Based on high expression levels (represented by TPM normalized counts) and cell cycle-related annotation of the transcripts we identified two potential effectors in *P. brassicae*; a mitotic checkpoint protein (BUB3) and a serine threonine kinase (AURKA) and in the *M. ectocarpii* transcriptome a putative Anaphase complex subunit 10 (ANAPC10) and a MOB kinase activator (MOB1).

## 4 Discussion

### 4.1 Local endoreduplication is a conserved mechanism during phytomyxid–host interaction

**Figure 4:**
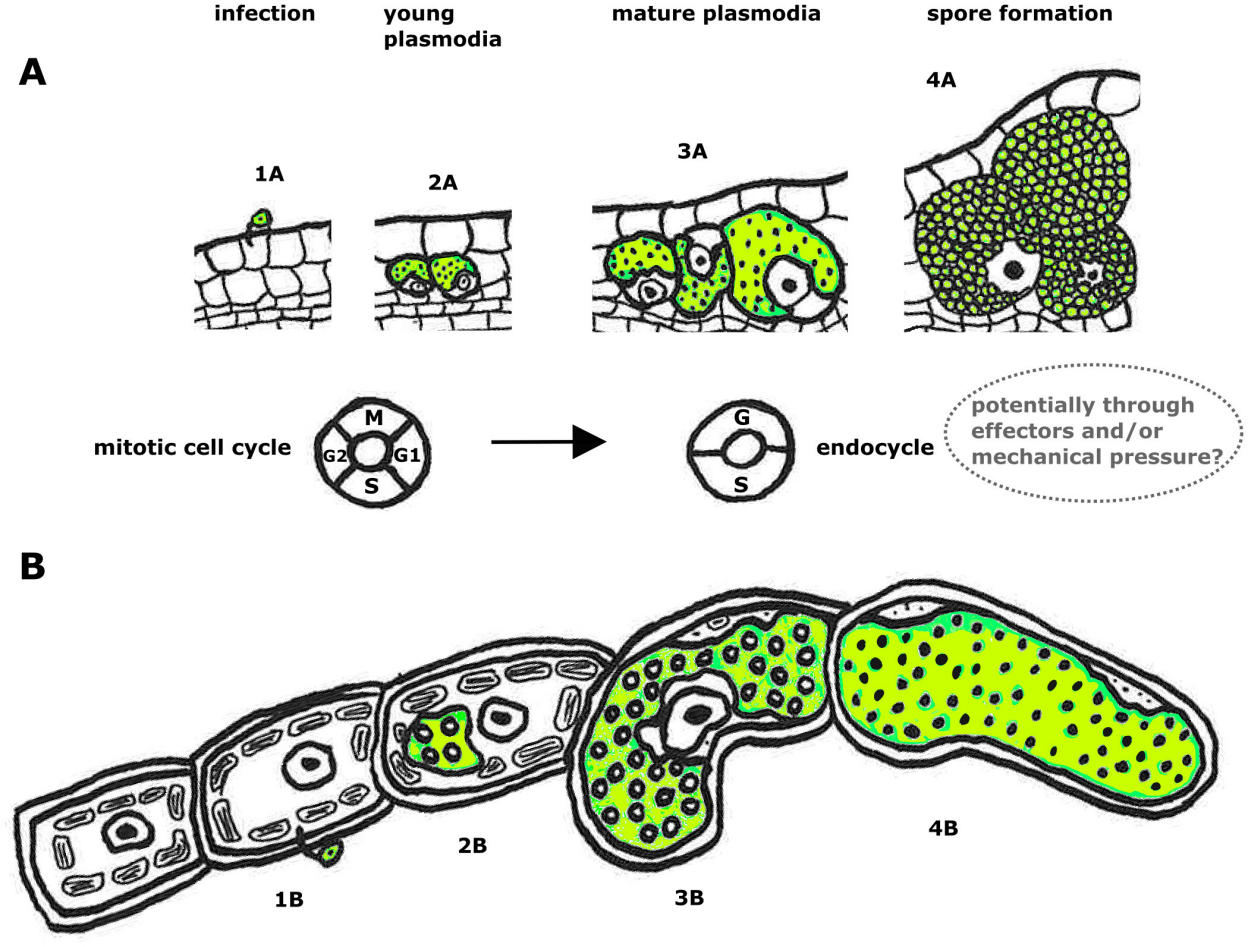
Schematic overview of how phytomyxean infection affects host cell and nucleus size [plant root **(A)** and brown algae **(B)**]. Infection process of *P. brassicae* in a plant root from infection with a zoospore (1A), development of multinucleate plasmodia (2A, 3A) to resting spore formation (4A). Below is the infection *of M. ectocarpii* in brown algae: 1B infection with a zoospore, development of multinucleate plasmodia (2B, 3B) to formation of a zoosporangium filled with zoospores (4B). The cell cycle machinery of the host switches during the progress of infection (at the plasmodial stage 3A, 3B when the host cells are getting hypertrophied) from the mitotic cell cycle to the endocycle. We hypothesize that phytomyxids may induce endoreduplication directly (through endocycle-inducing effectors), indirectly (as a host cell response to mechanical pressure from the growing intracellular plasmodium), or a combination of the two.

We demonstrate local endoreduplication in plant and stramenopile host cells colonized by phytomyxids, hinting at a central role of this altered physiological state of the host cells for the parasite (Figure 4). During phytomyxid infection, endoreduplication plays a crucial role in creating hypertrophied cells and consequently allows the parasite to use more space and create a nutrient sink for itself. Both the sporangial phase (*Maullinia ectocarpii*) and the sporogenic phase (*P. brassicae and M. braseltonii*) show endoreduplication, but the effect appears stronger during the sporogenic phase. Endoreduplication is an important mechanism involved in plant growth and development (Joubès and Chevalier, 2000; Lee et al., 2009), and changes to the plant endocycle are involved in the successful colonization of plants by biotrophic pathogens and mutualists (Carotenuto et al., 2019; De Almeida Engler et al., 2012). An increase in the size of host nuclei and cells is an indicator of active endoreduplication because DNA in the nucleus is multiplied, but the cells do not divide (Carotenuto et al., 2019; Sugimoto-Shirasu and Roberts, 2003). Measurements of the size of the nuclei, analysis of the ploidy of cells, and transcriptome data of infected material support our hypothesis that endoreduplication is induced by phytomyxids in their host and that this process is conserved in plants and brown algae. According to the presented findings, host endoreduplication is linked to the growth of all Phytomyxea, likely inducing an energy sink in the host that causes the energy transfer from the host to the parasite. This expands previous findings in *P. brassicae* to other Phytomyxea (Malinowski et al., 2019; Olszak et al., 2019).

By examining the changes in colonized host cells during the phytomyxid life cycle, it has been observed that the nucleus size of brown algae and plant cells increases following the growth of phytomyxid plasmodia (Figure 2). Endoreduplication is induced when a plasmodium colonizes a host cell and is maintained until the plasmodium differentiates into resting spores/zoospores (Figure 2), supporting the hypothesis that the induction of local endoreduplication is involved in generating a nutrient sink for phytomyxids (Malinowski et al., 2019). Cells that undergo endoreduplication show an increase in transcription and metabolic activity, making them an energy sink within the plant (Bourdon et al., 2012; Lang and Schnittger, 2020; Lee et al., 2009). This energy sink is exploited by Phytomyxea until they form resting spores (Figures 2D, H) when metabolic activity in the parasite is likely ceased. The nucleus is likely phagocytized by the phytomyxid shortly before the resting spores are differentiated (Garvetto et al., 2023). Exploiting of endoreduplication is therefore likely a common, and very basal feature of Phytomyxea–host interaction, as it is present in plants and brown algae.

### 4.2 Endoreduplication is a universal, and constant process during the Phytomyxid life cycle

Phytomyxea have two functionally different types of plasmodia: the sporangial (primary) plasmodia are formed during the initial phase of infection and can be found in main and alternative hosts (Neuhauser et al., 2014). Sporogenic (secondary) plasmodia are only found in main hosts (Neuhauser et al., 2011). The data we present indicate that both types of intracellular plasmodia trigger local endoreduplication in their respective host cells. Sporogenic plasmodia of *P. brassicae* and *M. braseltonii* showed 2.8 times enlarged nuclei in cells where multinucleate parasite plasmodia were present (Figures 1, 3). The sporangial plasmodia of *M. ectocarpii* induced a significant, yet smaller endoreduplication effect on the host than the closely related *M. braseltonii* or the plant colonizing *P. brassicae* (1.2 times enlarged nuclei, Figures 1, 3). Because of the limitations in obtaining infected material where both stages are present concurrently, we were unable to measure the effect of both sporangial and sporogenic plasmodia in the same host. Despite this limitation, different degrees of endoreduplication are linked to biological differences between the two plasmodial stages. The sporangial phase of the phytomyxid life cycle involves relatively short-lived and small plasmodia that colonize the root hairs (in plants) and filamentous thalli/gametophytes (in brown algae) of their host (Kageyama and Asano, 2009; Maier et al., 2000). In comparison, in *P. brassicae*, the completion of the sporogenic part of the life cycle takes ~20–40 days. The faster transition of sporangial plasmodia from infection to zoospore formation limits the number of endoreduplication cycles in the colonized host cell.

The sporogenic part of the phytomyxid life cycle is strongly linked to hypertrophy in the host, at the level of isolated cells, but also at the level of hypertrophied areas of tissue and macroscopic galls (Karling, 1968; Kolátková et al., 2021; Neuhauser et al., 2010). Infected cells in these galls are filled with large, multinucleate plasmodia with sometimes hundreds of nuclei, and it has been estimated that one large clubroot can contain billions of resting spores (Hwang et al., 2013; Liu et al., 2020). To produce such large amounts of spores and such large plasmodia, time and energy are needed for the parasite to grow, which is reflected in the longer duration of this part of the life cycle. It is unclear if the larger host nuclei in the sporogenic phase are due to a specialized interaction between phytomyxid and host (Figure 3); or if they are indirectly caused by the prolonged physical interaction period between plasmodia and host cells (Figure 4). Endoreduplication as a conserved feature of phytomyxid–host interaction could have different biological constraints or drivers. Larger plasmodia such as those seen during sporogenic growth can be interpreted as a result of the increased tolerance of specific tissues for endoreduplication (Bothwell J. H. et al., 2010; Garbary and Clarke, 2002). Tissues with greater tolerance for endoreduplication allow for larger, faster-growing cells, ultimately leading to more energy transfer and longer growth time for the parasite, which enables larger plasmodia formation.

### 4.3 Patterns of gene expression in the host support increased rates of endoreduplication during infection

The microscopic evidence of endoreduplication in infected host cells is supported by existing transcriptome data, suggesting that Phytomyxea induce endoreduplication via CCS52 and/or WEE1 kinase (Supplementary Table 1). In plants, the endocycle has been extensively studied and can be induced through different pathways including the activation of the APC/C and the consequent inactivation of mitotic cyclins through cell cycle switch proteins (CCS52) in leaves, trichomes, and roots (Cebolla et al., 1999; Heyman et al., 2017; Lammens et al., 2008); the activation of the WEE1 kinase in tomato fruit (Gonzalez et al., 2007); and the inhibition of CDKs by SIM/SMR proteins in leaves and trichomes (Kasili et al., 2010; Kumar et al., 2015). The molecular basis of endoreduplication in brown algae is less well understood (Bothwell J. H. et al., 2010; Garbary and Clarke, 2002). A variety of cell cycle switch proteins was upregulated in infected *B. oleracea subsp. gongylodes* and *B. rapa subsp. pekinensis* plants compared to the control plants (Supplementary Table 1) similar to a targeted study in *A. thaliana* (Olszak et al., 2019). A homolog of plant cell cycle switch proteins was differentially regulated in *E. siliculosus* infected with *M. ectocarpii*. The observed patterns are similar to findings of galls during biotrophic nematode infections (De Almeida Engler et al., 2012), or during arbuscular mycorrhiza symbiosis (Carotenuto et al., 2019), or in rhizobia-induced nodules in soybean (Fan et al., 2022). Transcriptional activation of the WEE1 kinase inhibits CDK activity, which subsequently induces endoreduplication (Gonzalez et al., 2007). The regulatory pathway involving the WEE1 kinase was also activated in plant hosts infected with phytomyxids (Supplementary Table 1) (Olszak et al., 2019). In *M. ectocarpii-*infected *E. siliculosus*, however, we detected a decrease in the expression of *WEE1* homologous transcripts. Endoreduplication induced by *WEE1* is very specific and appears to be restricted to specific tissues or organisms; for example, it controls endocycle onset in tomato fruit and maize endosperm, but not in *A. thaliana* leaves (Bhosale et al., 2019; De Veylder et al., 2011). However, the role of WEE1 has not been validated in brown algae and may differ. In mammals, for example, WEE1 regulates the cell cycle and is important during DNA damage checkpoints (Elbæk et al., 2020). WEE1 may also be involved in DNA checkpoint control in *Arabidopsis* during nematode infection (Cabral et al., 2020).

### 4.4 Possible scenarios behind Phytomyxea-driven endocycle stimulation

The mechanism behind the Phytomyxea-induced endocycle is still unknown (Malinowski et al., 2019; Olszak et al., 2019) but our findings allow for a more comprehensive debate. Phytomyxea can induce endoreduplication either actively via effector molecules that target the cell cycle machinery or passively through the mechanical stress/tension caused by the growing plasmodium (Toruño et al., 2016). Biotrophs, such as arbuscular mycorrhiza fungi, powdery mildew, nematodes, and bacteria including rhizobia are known to use effectors to manipulate the host cells (Goverse and Smant, 2014; Rafiqi et al., 2012). Effectors are key to understanding the pathogen–host interaction, therefore potential effectors of *P. brassicae* are well-studied (Muirhead and Pérez-López, 2022; Pérez-López et al., 2020; Rolfe et al., 2016). Our search for effector candidates that interact with cell cycle proteins identified two putative effectors in *P. brassicae* and *M. ectocarpii*, respectively. Those potential effector candidates together with the results of Pérez-López et al. (2020), who identified a *P. brassicae* cyclin as a putative effector, highlight the potential of effectors in manipulating the host cell cycle. This makes putative effectors targeting host processes related to the induction of the endocycle promising targets for future studies to better understand phytomyxid–host interaction.

The second viable hypothesis for the induction of endoreduplication is mechanic stress caused by the growth of the intracellular parasite, indirectly keeping the cells “locked” in the endocycle stage until the plasmodium produces spores. Cells in the root cortex undergo one or two rounds of endoreduplication during normal root growth (Bhosale et al., 2019). Physical activation of the endocycle has been suggested as the mechanism for biotrophic nematodes (de Almeida Engler and Gheysen, 2013). It is also discussed that *P. brassicae* induces wall stress and cell expansion in its host (Badstöber et al., 2020b), resulting in alterations in the cell cycle (Olszak et al., 2019). Endoreduplication is linked to cell growth and cell wall remodeling (Bhosale et al., 2019; Bhosale and Vissenberg, 2023), so the growth of the parasite could indirectly induce the host to undergo additional rounds of endocycling, with the resulting energy sink providing nutrients for the Phytomyxea. The regulation and induction of endoreduplication in brown algae are even less clear, as studies on the topic are scant (Bothwell J. H. et al., 2010; Garbary and Clarke, 2002). However, based on the findings discussed here, we hypothesize that similar active and passive processes could be involved in the induction of endoreduplication in all hosts of Phytomyxea.

Endoreduplication plays a role in cell growth, and ploidy level often correlates with cell size (Breuer et al., 2007; Chevalier et al., 2011; Sugimoto-Shirasu and Roberts, 2003). Increased size of the host cells provides space for the growth of the plasmodia, as demonstrated by galls formed in *A. thaliana ccs52a1* mutants with deficient endoreduplication, which were found to be significantly smaller than those of wild-type *A. thaliana* (Olszak et al., 2019). Therefore, the increased metabolic activity of the host allows for higher energy transfer and combined with larger host cells provides more space and energy to be translated into the growth of the phytomyxid parasites.

## 5 Conclusion

In this study, we demonstrate that locally induced endoreduplication is a conserved response of the host upon infection with Phytomyxea. Therefore, the parasite benefits directly from this alteration in the physiology of the host cell, either from accessing additional nutrients, from gaining more growth space, or, likely, from both. The exact nature of this interaction is yet to be determined. However, it can be hypothesized that the host's ability to tolerate endoreduplication is linked to the ability of the pathogen to grow and propagate. One possible scenario is, that the growth of the intracellular phytomyxid plasmodium has an indirect impact on the host cell growth, where mechanical force results in the induction and maintenance of the cell in endocycle. The alternative scenario explaining the endocycle of infected host cells could be based on an active process of manipulation and resource negotiation between the host and phytomyxid. The conserved nature of endoreduplication in host cells infected with phytomyxids is an important jigsaw piece to understanding phytomyxid–host interaction because cells undergoing endoreduplication generate an energy sink reallocating nutrients to cells infected with the parasite and are therefore at the basis of this enigmatic biotrophic interaction.

## Data Availability

The original contributions presented in the study are included in the article/Supplementary material, further inquiries can be directed to the corresponding author.
