# Supplementary material for: Local endoreduplication of the host is a conserved process during Phytomyxea–host interaction
Source: Front Microbiol. 2025 Feb 5;15:1494905. doi: 10.3389/fmicb.2024.1494905 (PMC11835965; doi:10.3389/fmicb.2024.1494905)
Supplement: Supplementary file 1 [file Data_Sheet_1.docx]

**SUPPLEMENTARTY MATERIAL**

**Supplementary Figures 1- 6**

**Supplementary Tables 1-7**

**Supplementary Results**

**Supplementary Methods**


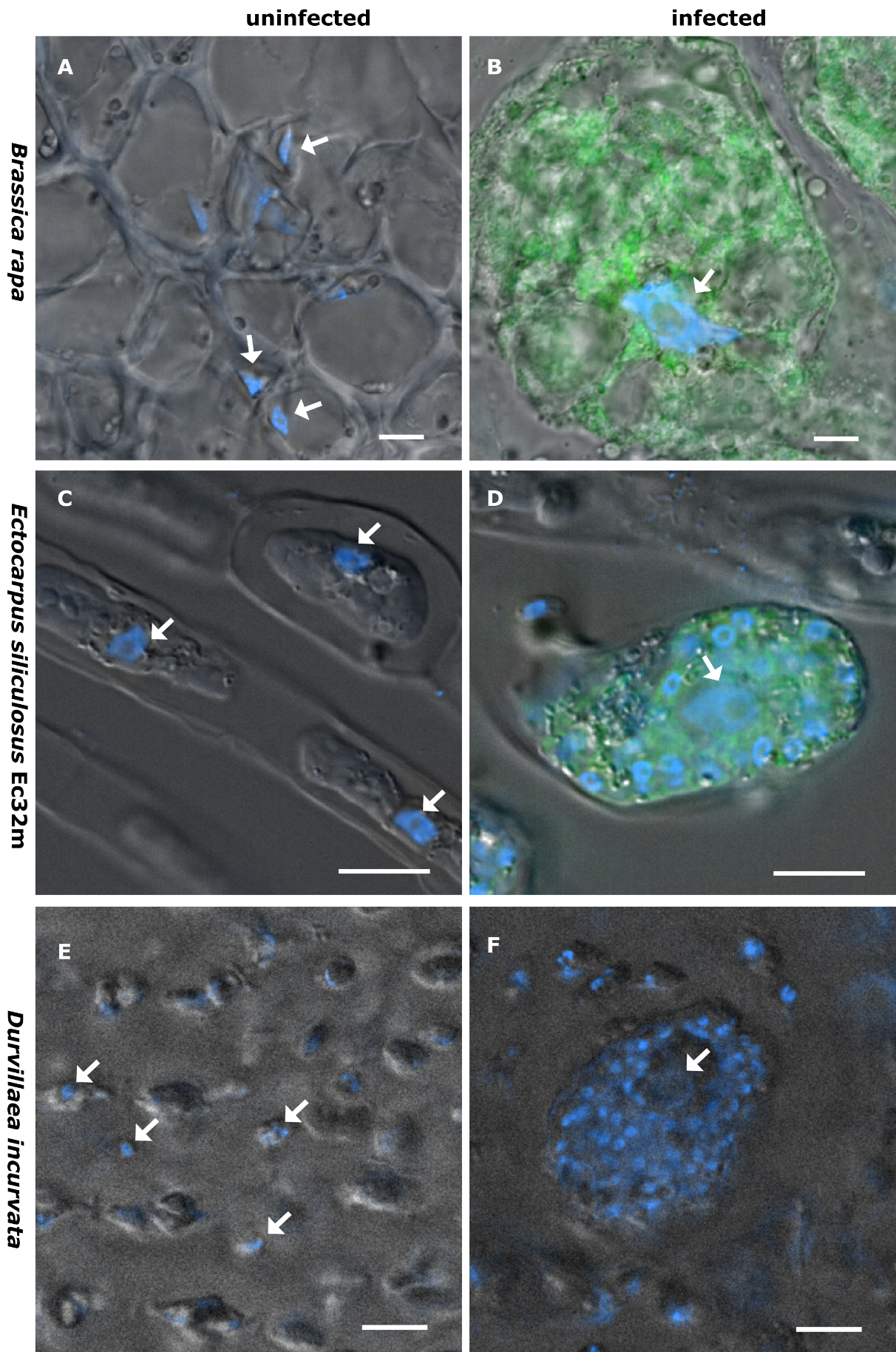


**Supplementary Figure 1. Nucleus size and shape vary between infected and non-infected hosts.** Uninfected *Brassica rapa subsp. pekinensis* (a), plasmodium of *Plasmodiophora brassicae* in *B. rapa subsp. pekinensis* (b), uninfected *Ectocarpus siliculosus* Ec32m (c), multinucleate plasmodium of *Maullinia ectocarpii* in *E. siliculosus* Ec32m (d), uninfected *Durvillaea incurvata* (e), and multinucleate plasmodium of *Maullinia braseltonii* in *D. incurvata* (f). Overlay of DIC image, Hoechst and FISH (a, b, b, d); overlay of DIC image and Hoechst (e, f). Scale bar: 10µm.


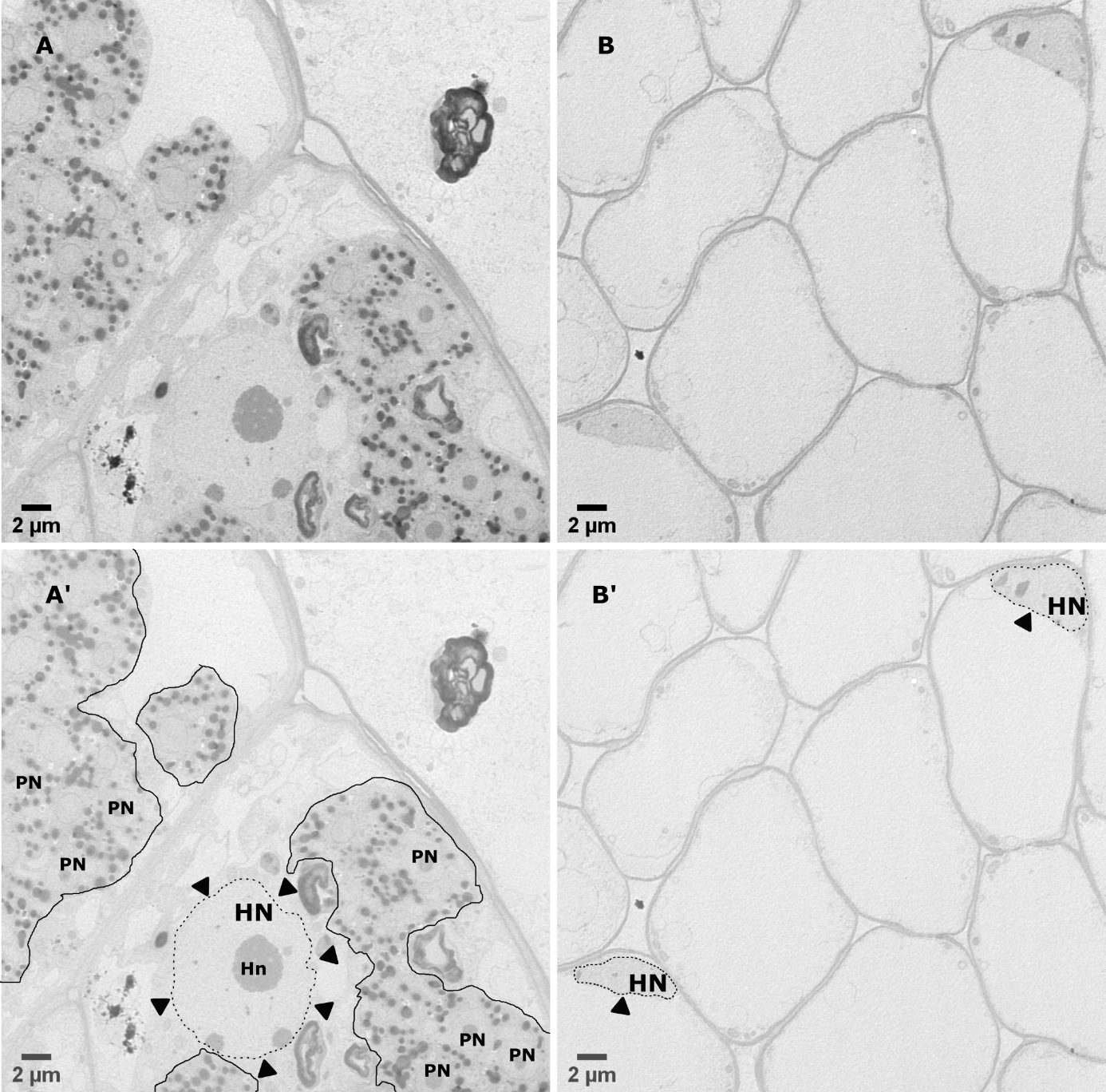


**Supplementary Figure 2. Transmission electron microscopy (TEM) of *Plasmodiophora brassicae* infected *Brassica rapa subsp. pekinensis* and uninfected *B. rapa subsp. pekinensis* root cells.** Infected host cell with an intact enlarged host nucleus (arrowhead) surrounded by the plasmodium of *P. brassicae* (a, a’). Uninfected root cell of *B. rapa subsp. pekinensis* shows a “normal” sized host nucleus (b, b’). Scale bar 2 µm. HN Host nucleus, Hn Host nucleolus, PN parasite nucleus.


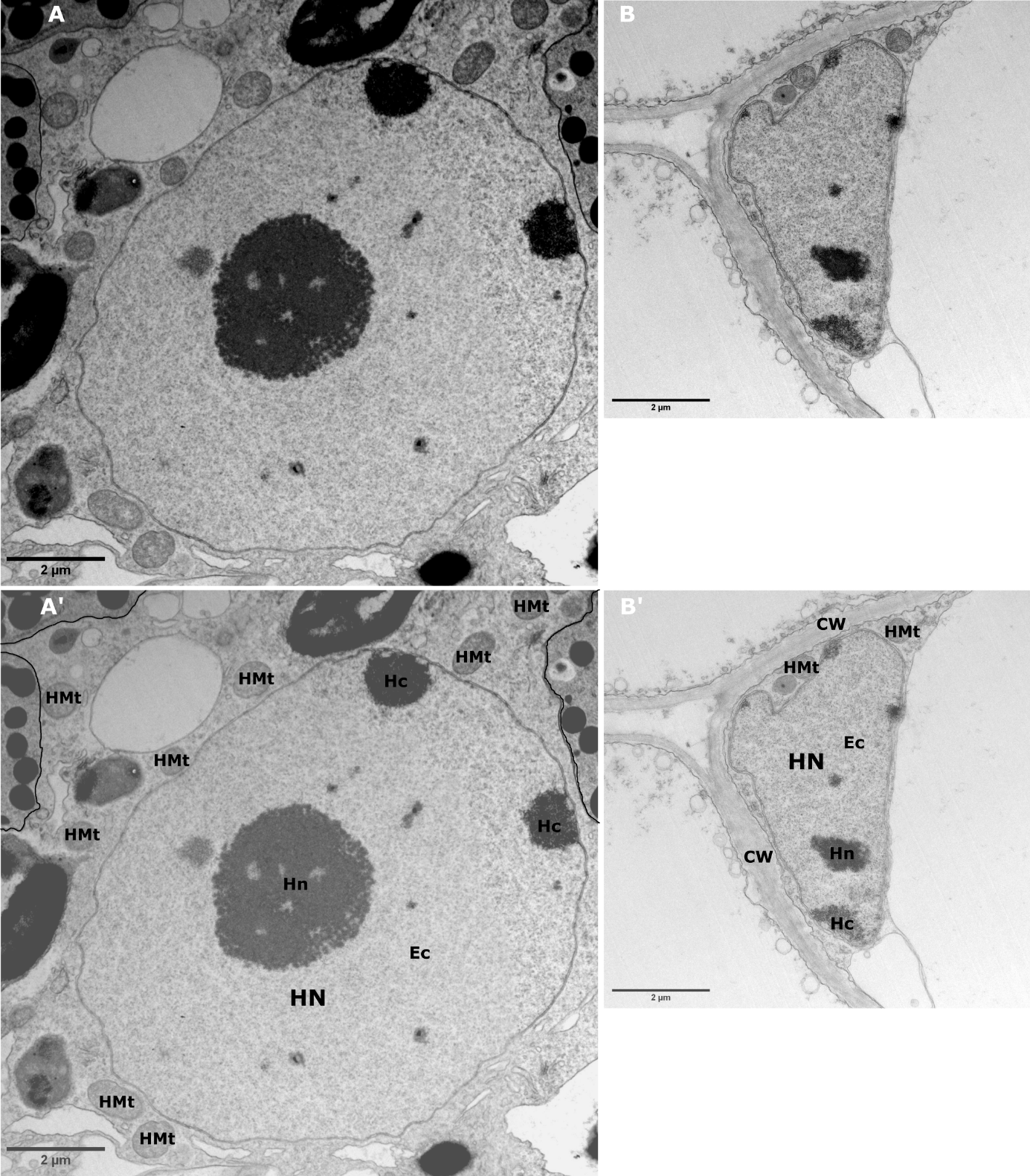


**Supplementary Figure 3. Transmission electron microscopy (TEM) of *Plasmodiophora brassicae* infected *Brassica rapa subsp. pekinensis* and uninfected *B. rapa subsp. pekinensis* root cells.** Infected host cell with an intact enlarged host nucleus (arrowhead) surrounded by the plasmodium of *P. brassicae* (a, a’). Uninfected root cell of *B. rapa subsp. pekinensis* shows a “normal” sized host nucleus (b, b’). Scale bar 2 µm. HN Host nucleus, Hn Host nucleolus, Ec Euchromatin, Hc Heterochromatin, HMt Host mitochondrion, CW cell wall


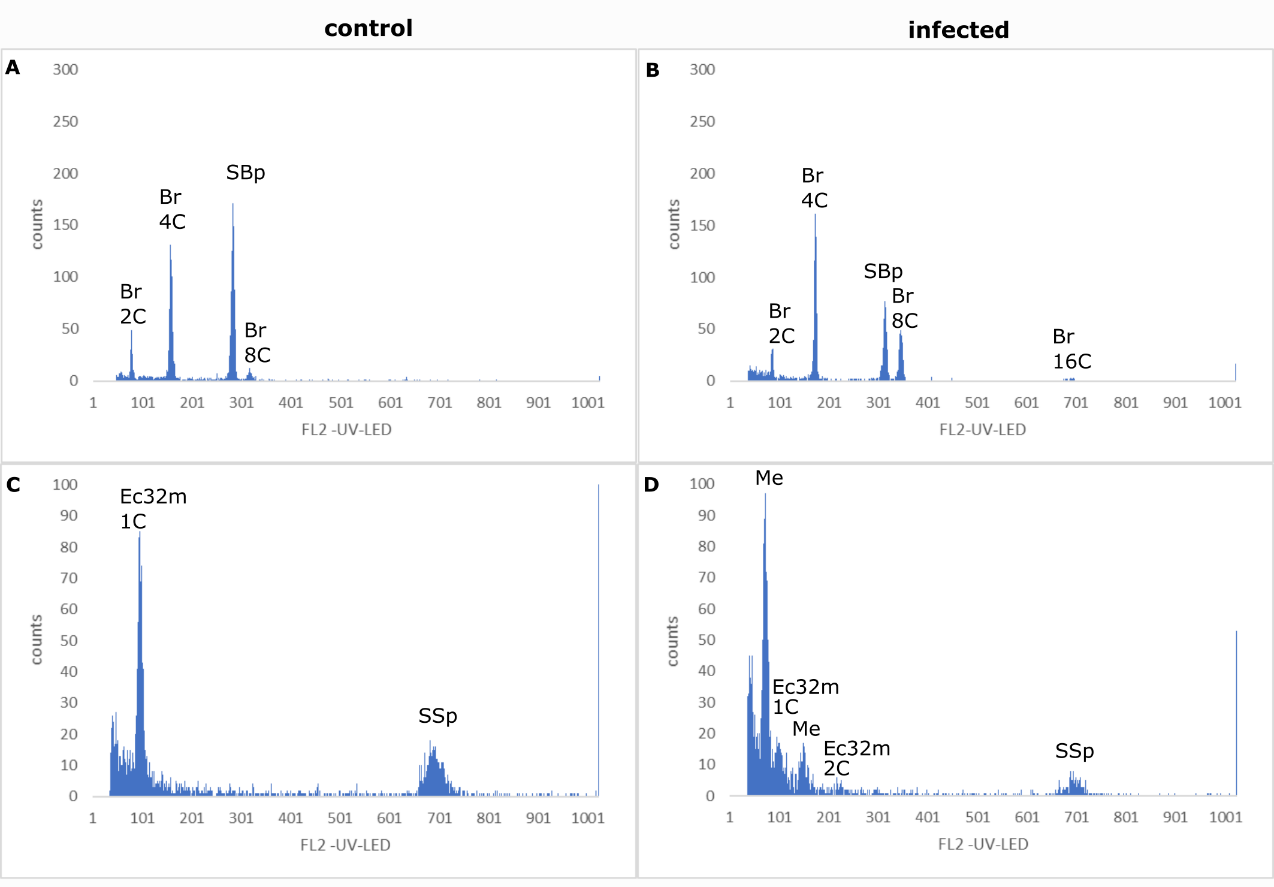


**Supplementary Figure 4.** Histogram of relative DNA content from flow cytometry data of roots from uninfected plants (*Brassica rapa*) (a), *Plasmodiophora brassicae* infected *Brassica rapa* roots (b), uninfected *Ectocarpus siliculosus* Ec32m cultures (c), and *Maullinia ectocarpii* infected *Ectocarpus siliculosus* Ec32m cells (d). B.r…*Brassica rapa,* SBp…standard (*Bellis perennis*), Ec32m…*Ectocarpus siliculosus* Ec32m, Me…*Maullinia ectocarpii,* SSp…standard (*Solanum pseudocapsicum*).


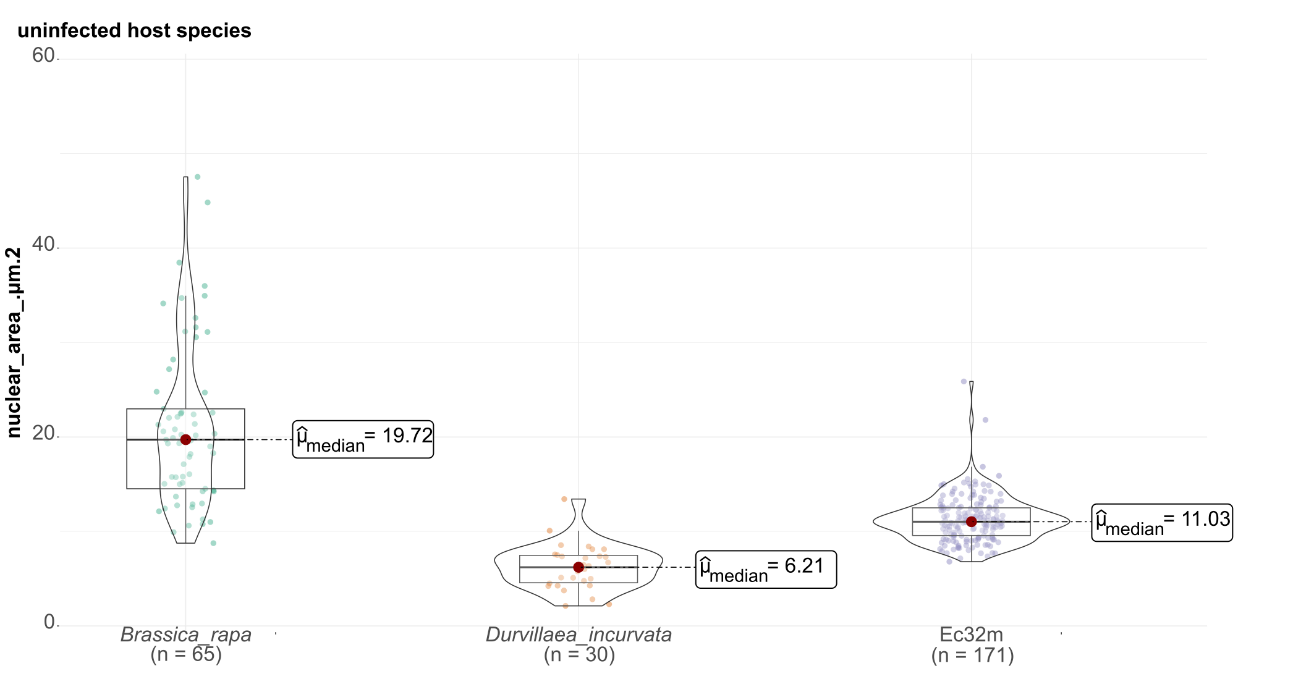


**Supplementary Figure 5.** **Nuclear size of uninfected hosts.** Distribution of nuclear area of *Brassica rapa*, *Durvillaea incurvata*, and *Ectocarpus siliculosus* Ec32m.


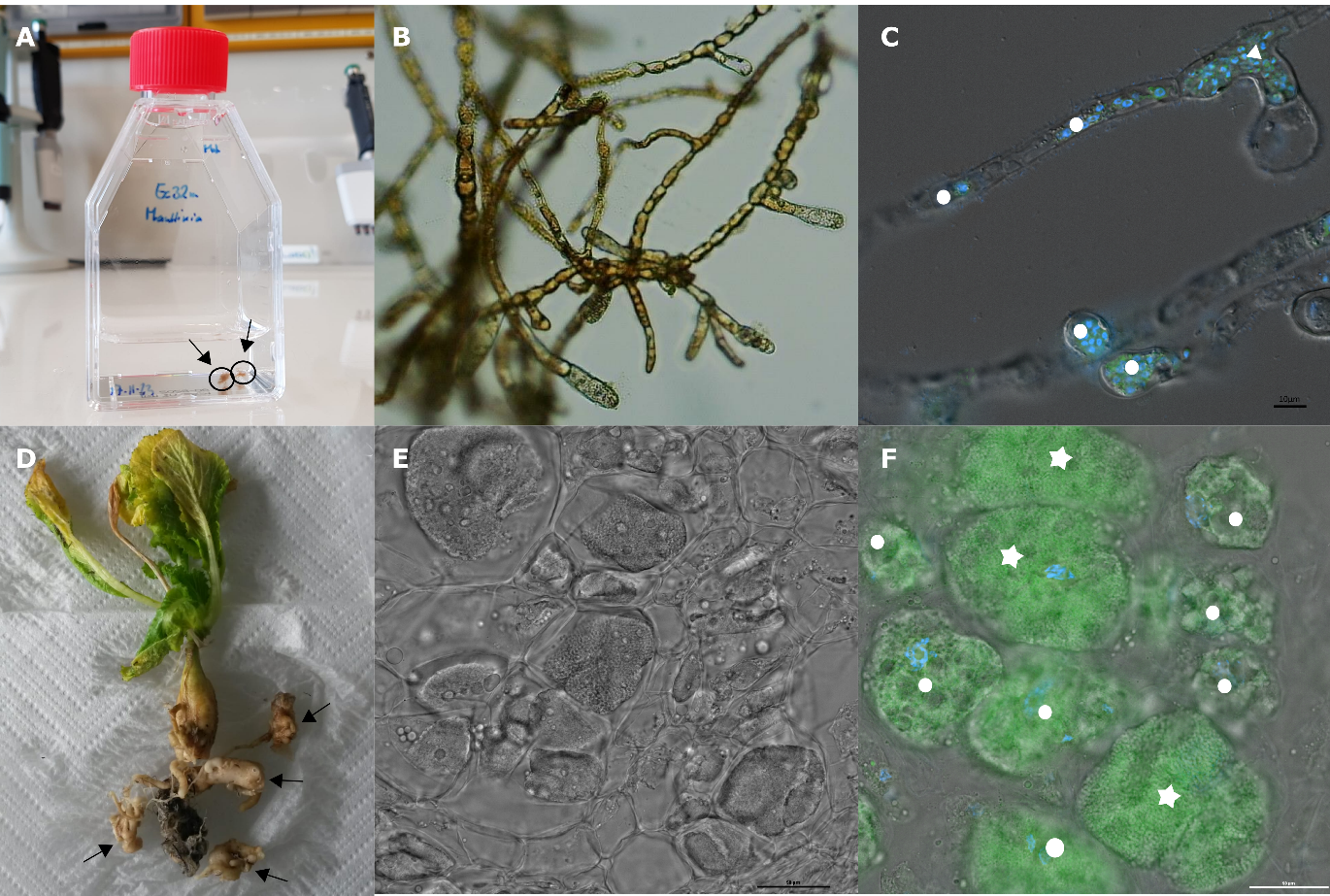


**Supplementary Figure 6.** **Phytomyxid infections.** Cultures of infected *Ectocarpus siliculosus* Ec32m (highlighted with arrows, inside circles) (a), filaments of *Maullinia ectocarpii* infected *Ectocarpus siliculosus* Ec32m (b) and *Ectocarpus siliculosus* Ec32m infected with different stages of *Maullinia ectocarpii*: plasmodia (filled circles) and zoosporangium filled with zoospores (triangle). *Brassica rapa* with clubroots (arrows) (c) section of a clubroot (e) *Brassica rapa* root cells filled with plasmodia (filled circles) and resting spores (stars) of *Plasmodiophora brassicae* (f). Overlay of DIC image, Hoechst and FISH (c, f).

**Supplementary Table1. Changes in activity of cell cycle genes during phytomyxean infection in plant endocycle and brown algae.** The genes were detected based on prior literature, particularly when they were linked to biotrophic interactions. Arrows indicate up- or down-regulation of the transcripts in Phytomyxea infected plants and algae. Underscore (_) indicates no changes in expression levels. Lack of any symbol indicate absence of the correspondent gene from the dataset. * based on FPKM instead of log2FC; ° (gene names from (Bothwell et al., 2010) and the OrcAE database); °° based on (De Almeida Engler et al., 2012; Gonzalez et al., 2007; Huysman et al., 2015; Inzé and De Veylder, 2006; Joubès and Chevalier, 2000; Lammens et al., 2008; Olszak et al., 2019). 1 =Data for *B. rapa var. pekinensis* from (Jia et al., 2017).

| *E. siliculosus* / *M. ectocarpii* | | *B. oleracea / P. brassicae* | | *B. rapa / P. brassicae^1^* | |  |  |  |
| --- | --- | --- | --- | --- | --- | --- | --- | --- |
| **Gene°** | **u/d** | **At homolog /ortholog** | **u/d** | **At homolog /ortholog** | **u/d** | **Previously described transcriptional changes °°** | **Function (in plants)** | **Behaviour of the gene in biotrophic interactions** |
| CDH1-Ccs52 (Ectsi FZR1) | ↑ | CCS52A1 | ↑* | CCS52A1 | ↑ | ↑ | CCS52A1 activator of APC/C; promotes endocycle (Larson-Rabin et al., 2009) | upregulated during nematode infection to promote endoreduplication (De Almeida Engler et al., 2012); upregulated during powdery mildew infection (Chandran and Wildermuth, 2016); homologs of CCS52A are active during rhizobia infection in soybean causing endoreduplication (Fan et al., 2022); Upregulated during ***P. brassicae*** infection in *A. thaliana* (Olszak et al., 2019) |
|  |  | CCS52A2 |  | CCS52A2 | ↓ | ↑ | CCS52A2 activator of APC/C; promotes endocycle (Lammens et al., 2008) | upregulated during nematode infection to promote endoreduplication (De Almeida Engler et al., 2012); homologs of CCS52A are active during rhizobia infection in soybean causing endoreduplication (Fan et al., 2022) |
|  |  | CCS52B | ↑ | CCS52B |  | ↑ | CCS52B activator of APC/C; promotes endocycle (De Almeida Engler et al., 2012) | upregulated during nematode infection to promote endoreduplication (De Almeida Engler et al., 2012) |
| wee1 (Ectsi Wee1) | ↓ | WEE1 | ↑ | WEE1 | ↑ | ↑ | WEE1 promotes endocycle in tomato fruit (Gonzalez et al., 2007) | upregulated during ***P. brassicae*** infection in *A. thaliana* (Olszak et al., 2019) |
| Ectsi CDKA1 | ↑ | CDKA1 | ↑* | CDKA1 |  | ? | control of mitotic cell cycle: G1/S and G2/M (Hemerly et al., 1995; Inzé and De Veylder, 2006) | downregulated during ***P. brassicae*** infection in *A. thaliana* (Olszak et al., 2019) |
| Ectsi CDKA2/CDKB | ↓ | CDKB1 (;1&2) | ↑_ | CDKB1 (;1&2) | ↑↑ | ↓ | CDKB1 controls the mitotic cell cycle: G2/M phase specific; negatively regulates the endocycle (Boudolf et al., 2006, 2004; Huysman et al., 2015; Porceddu et al., 2001) | Up and down regulated during ***P. brassicae*** infection in *A. thaliana* (Olszak et al., 2019) |
|  |  | CDKB2(;1&2) | ↑ | CDKB2(;1&2) | ↑↑ | ↓ | control of mitotic cell cycle: G2/M phase specific (transition) (Andersen et al., 2008; Menges et al., 2005) | downregulated during ***P. brassicae*** infection in *A. thaliana* (Olszak et al., 2019) |
| Ectsi CDKB ⁄ 4-like | ↑ |  |  |  |  |  |  | - |
| Ectsi E2F | ↑ | E2FA | ↑* | E2FA | ↑ | ↑ | E2Fa promotes G1-S transition (Ramirez‐Parra et al., 2003; Vandepoele et al., 2005) | upregulated during ***P. brassicae*** infection in *A. thaliana* (Olszak et al., 2019) |

**Supplementary Table 2.** Cell cycle genes differentially expressed in *Plasmodiophora brassicae* infected *Brassica oleracea* in comparison to noninfected *Brassica oleracea*. White galls where used for the comparison with the noninfected *Brassica* roots. Upregulated genes are highlighted with an upward arrow (↑), downregulated genes with a downward arrow (↓). Log2fold changes are colour coded (colour gradient from highest upregulated in green to downregulated in red). Genes important for endoreduplication are highlighted in blue.

| Gene | Query | Hit/Gene ID | log2FC | up/down |
| --- | --- | --- | --- | --- |
| CCS52A1 | AT4G22910 | TRINITY_DN89594_c1_g1_i2 | NA | ↑ |
| CCS52A2 | AT4G11920 |  |  |  |
| CCS52B | AT5G13840 | TRINITY_DN67752_c0_g2_i1 | 7.66 | ↑ |
| WEE1 | AT1G02970 | TRINITY_DN103154_c0_g1_i1 | 2.18 | ↑ |
| CDKA;1 | AT3G48750 | TRINITY_DN97304_c1_g1_i1 | NA | ↑ |
| CDKB1;1 | AT3G54180 | TRINITY_DN83815_c0_g1_i1 | 10.71 | ↑ |
| CDKB1;2 | AT2G38620 |  |  |  |
| CDKB2;1 | AT1G76540 | TRINITY_DN100092_c5_g6_i1 | 3.59 | ↑ |
| CDKB2;2 | AT1G20930 | TRINITY_DN100092_c0_g1_i1 | 6.19 | ↑ |
| CDKC;1 | AT5G10270 | TRINITY_DN97810_c3_g2_i1 | NA | ↓ |
| CDKD;1 | AT1G73690 |  |  |  |
| CDKD;3 | AT1G18040 | TRINITY_DN98599_c2_g1_i2 | 3.20 | ↑ |
| CDKF;1 | AT4G28980 | TRINITY_DN95900_c0_g1_i1 | NA | ↑ |
| CKS1 | AT2G27960 |  |  |  |
| CKS2 | AT2G27970 | TRINITY_DN108344_c0_g1_i1 | 3.37 | ↑ |
| CYCA1;1 | AT1G44110 | TRINITY_DN97779_c1_g1_i4 | 2.26 | ↑ |
| CYCA1;2 | AT1G77390 | TRINITY_DN79679_c0_g2_i1 | NA | ↑ |
| CYCA2;1 | AT5G25380 |  |  |  |
| CYCA2;2 | AT5G11300 | TRINITY_DN50245_c0_g3_i1 | 2.37 | ↑ |
| CYCA2;3 | AT1G15570 | TRINITY_DN99644_c2_g2_i2 | 7.95 | ↑ |
| CYCA2;4 | AT1G80370 | TRINITY_DN99644_c2_g3_i8 | 1.85 | ↑ |
| CYCB1;1 | AT4G37490 | TRINITY_DN98655_c3_g8_i1 | 2.35 | ↑ |
| CYCB1;2 | AT5G06150 | TRINITY_DN99804_c2_g1_i1 | 9.33 | ↑ |
| CYCB1;3 | AT3G11520 | TRINITY_DN99804_c3_g1_i1 | 4.07 | ↑ |
| CYCB1;4 | AT2G26760 |  |  |  |
| CYCB2;1 | AT2G17620 | TRINITY_DN35954_c0_g2_i1 | NA | ↑ |
| CYCB2;2 | AT4G35620 | TRINITY_DN92473_c1_g2_i1 | NA | ↑ |
| CYCB2;3 | AT1G20610 | TRINITY_DN75673_c0_g2_i1 | NA | ↑ |
| CYCB2;4 | AT1G76310 | TRINITY_DN64261_c0_g1_i1 | 3.54 | ↑ |
| CYCB3;1 | AT1G16330 | TRINITY_DN88505_c1_g1_i2 | 8.19 | ↑ |
| CYCD1;1 | AT1G70210 | TRINITY_DN84213_c1_g2_i1 | 2.83 | ↑ |
| CYCD2;1 | AT2G22490 | TRINITY_DN94583_c1_g1_i1 | 1.62 | ↑ |
| CYCD3;1 | AT4G34160 | TRINITY_DN93529_c0_g2_i6 | 4.25 | ↑ |
| CYCD3;2 | AT5G67260 | TRINITY_DN3146_c0_g1_i1 | NA | ↑ |
| CYCD3;3 | AT3G50070 | TRINITY_DN70625_c0_g1_i1 | 1.58 | ↑ |
| CYCD5;1 | AT4G37630 | TRINITY_DN30164_c0_g2_i1 | 1.78 | ↑ |
| CYCD6;1 | AT4G03270 | TRINITY_DN3457_c1_g1_i1 | 2.66 | ↑ |
| DPa | AT5G02470 | TRINITY_DN98378_c3_g1_i2 | NA | ↑ |
| E2Fa | AT2G36010 | TRINITY_DN99974_c0_g1_i8 | NA | ↑ |
| E2Fc | AT1G47870 | TRINITY_DN2252_c1_g1_i1 | NA | ↑ |
| E2Fd/DEL2 | AT5G14960 | TRINITY_DN63323_c0_g1_i1 | 2.51 | ↑ |
| E2Ff/DEL3 | AT3G01330 | TRINITY_DN83317_c0_g1_i1 | 6.78 | ↑ |
| DEL1 | AT3G48160 | TRINITY_DN91414_c0_g1_i2 | 6.0502 | ↑ |
| KRP1 | AT2G23430 |  |  |  |
| KRP3 | AT5G48820 | TRINITY_DN93265_c1_g2_i1 | NA | ↑ |
| KRP5 | AT3G24810 | TRINITY_DN66009_c0_g1_i1 | NA | ↓ |
| KRP7 | AT1G49620 |  |  |  |
| MYB3R1 | AT4G32730 | TRINITY_DN100421_c0_g4_i1 | 5.17 | ↑ |
| MYB3R4 | AT5G11510 | TRINITY_DN92052_c1_g1_i1 | 1.93 | ↑ |
| SIM | AT5G04470 | TRINITY_DN47290_c1_g1_i1 | NA | ↓ |
| SMR1 | AT3G10525 | TRINITY_DN89587_c0_g1_i1 | NA | ↓ |
| SMR10 | AT2G28870 | TRINITY_DN77455_c0_g2_i1 | -1.55 | ↓ |
| SMR11 | AT2G28330 |  |  |  |
| SMR13 | AT3G20898 |  |  |  |
| SMR14 | AT5G59360 |  |  |  |
| SMR2 | AT1G08180 | TRINITY_DN159569_c1_g1_i1 | -1.59 | ↓ |
| SMR3 | AT5G02420 |  |  |  |
| SMR6 | AT5G40460 |  |  |  |
| SMR8 | AT1G10690 | TRINITY_DN90258_c0_g1_i1 | -1.56 | ↓ |
| SMR9 | AT1G51355 |  |  |  |
| RBR1 | AT3G12280 | TRINITY_DN97437_c1_g3_i2 | 1.39 | ↑ |
| CDC20 | AT4G33270 | TRINITY_DN94967_c0_g1_i3 | 7.61 | ↑ |
| APC1 | AT5G05560 | TRINITY_DN99913_c2_g1_i2 | NA | - |
| APC2 | AT2G04660 | TRINITY_DN99749_c1_g2_i4 | NA | ↑ |
| APC6 | AT1G78770 | TRINITY_DN69002_c0_g1_i1 | 1.12 | ↑ |

**Supplementary Table 3.** Cell cycle genes differentially expressed in *Plasmodiophora brassicae* infected susceptible *Brassica rapa subsp. pekinensis* in comparison to noninfected susceptible *Brassica rapa subsp. pekinensis* (from Jia et al., 2017). Upregulated genes are highlighted with an upward arrow (↑), downregulated genes with a downward arrow (↓). Log2fold changes are colour coded (colour gradient from highest upregulated in green to downregulated in red). Genes important for endoreduplication are highlighted in blue.

| Gene | Query | Gene ID in *B.rapa* genome | log2FC | up/down |
| --- | --- | --- | --- | --- |
| CCS52A1 | AT4G22910 | Bra019347 | 2,45629 | ↑ |
| CCS52A2 | AT4G11920 | Bra029426 | -2,6299 | ↓ |
| CCS52B | AT5G13840 | Bra006219 |  |  |
| WEE1 | AT1G02970 | Bra032577 | 1,23237 | ↑ |
| CDKA;1 | AT3G48750 | Bra029917/Bra018036 |  |  |
| CDKB1;1 | AT3G54180 | Bra014835 | 1,93967 | ↑ |
| CDKB1;2 | AT2G38620 | Bra000077 | 3,25573 | ↑ |
| CDKB2;1 | AT1G76540 | Bra015735 | 1,70825 | ↑ |
| CDKB2;2 | AT1G20930 | Bra012258 | 2,32092 | ↑ |
| CDKC;1 | AT5G10270 | Bra009045 |  |  |
| CDKD;1 | AT1G73690 | Bra015974 |  |  |
| CDKD;3 | AT1G18040 | Bra016572/Bra031013 |  |  |
| CDKF;1 | AT4G28980 | Bra011073 |  |  |
| CKS1 | AT2G27960 | Bra034614 |  |  |
| CKS2 | AT2G27970 | Bra034380 | 1,93691 | ↑ |
| CYCA1;1 | AT1G44110 | Bra032202 | 2,91266 | ↑ |
| CYCA1;2 | AT1G77390 | Bra015671/Bra022173 |  |  |
| CYCA2;1 | AT5G25380 | NA |  |  |
| CYCA2;2 | AT5G11300 | Bra008973 | 2,41315 | ↑ |
| CYCA2;3 | AT1G15570 | Bra026120 | 1,96338 | ↑ |
| CYCA2;4 | AT1G80370 | Bra035186 | 2,31131 | ↑ |
| CYCB1;1 | AT4G37490 | Bra011769 | 2,96905 | ↑ |
| CYCB1;2 | AT5G06150 | Bra028741 | 1,43761 | ↑ |
| CYCB1;2 | AT5G06150 | Bra005880 | 1,36923 | ↑ |
| CYCB1;3 | AT3G11520 | Bra002046 | 2,09866 | ↑ |
| CYCB1;4 | AT2G26760 | NA |  |  |
| CYCB2;1 | AT2G17620 | Bra037265 | 1,55631 | ↑ |
| CYCB2;2 | AT4G35620 | Bra017726 | 1,5446 | ↑ |
| CYCB2;3 | AT1G20610 | Bra016460 | 2,82729 | ↑ |
| CYCB2;4 | AT1G76310 | Bra015762 | 1,65451 | ↑ |
| CYCB2;4 | AT1G76310 | Bra003727 | 1,02333 | ↑ |
| CYCB3;1 | AT1G16330 | Bra026065 | 1,62325 | ↑ |
| CYCB3;1 | AT1G16330 | Bra016640 | 1,73046 | ↑ |
| CYCD1;1 | AT1G70210 | Bra007901 | 2,08892 | ↑ |
| CYCD2;1 | AT2G22490 | Bra038518 | 2,17862 | ↑ |
| CYCD2;1 | AT2G22490 | Bra030232 | 3,03377 | ↑ |
| CYCD3;1 | AT4G34160 | Bra011501 | 3,28156 | ↑ |
| CYCD3;1 | AT4G34160 | Bra034612 | 1,62723 | ↑ |
| CYCD3;2 | AT5G67260 | Bra012146 |  |  |
| CYCD3;3 | AT3G50070 | Bra036051 | 1,27954 | ↑ |
| CYCD5;1 | AT4G37630 | Bra017840 | 1,46259 | ↑ |
| CYCD6;1 | AT4G03270 | Bra034198 | 2,39634 | ↑ |
| DPa | AT5G02470 | Bra005726 | 2,67388 | ↑ |
| E2Fa | AT2G36010 | Bra005305 | 1,78689 | ↑ |
| E2Fc | AT1G47870 | Bra014096 | 1,41388 | ↑ |
| E2Fd/DEL2 | AT5G14960 | Bra023497 | 2,60637 | ↑ |
| E2Ff/DEL3 | AT3G01330 | Bra039127 |  |  |
| DEL1 | AT3G48160 | Bra033767 | 9,582 | ↑ |
| KRP1 | AT2G23430 | Bra039199 |  |  |
| KRP3 | AT5G48820 | Bra037459/Bra036159/Bra010440 | |  |
| KRP5 | AT3G24810 | Bra013240 | 3,22642 | ↑ |
| KRP7 | AT1G49620 | Bra018812 | 1,80071 | ↑ |
| MYB3R1 | AT4G32730 | Bra037045 | 1,46859 | ↑ |
| MYB3R1 | AT4G32730 | Bra011376 | 1,75858 | ↑ |
| MYB3R4 | AT5G11510 | Bra006102/Bra008956 |  |  |
| SIM | AT5G04470 | Bra005808/Bra028788/Bra009451 | |  |
| SMR1 | AT3G10525 | Bra029848 | -1,9529 | ↓ |
| SMR10 | AT2G28870 | Bra000479 | -1,36965 | ↓ |
| SMR11 | AT2G28330 | Bra011957/Bra011958 |  |  |
| SMR13 | AT3G20898 | Bra023914 | -2,56272 | ↓ |
| SMR13 | AT3G20898 | Bra001805 | -1,16122 | ↓ |
| SMR13 | AT3G20898 | Bra031241 | -1,54695 | ↓ |
| SMR14 | AT5G59360 | Bra002572 |  |  |
| SMR2 | AT1G08180 | Bra030715 | -1,12688 | ↓ |
| SMR3 | AT5G02420 | Bra005723 | -3,8575 | ↓ |
| SMR3 | AT5G02420 | Bra009590 | -1,96998 | ↓ |
| SMR6 | AT5G40460 | Bra025573 | 1,42995 | ↑ |
| SMR8 | AT1G10690 | Bra019906 | -2,60225 | ↓ |
| SMR9 | AT1G51355 | Bra014270 | 2,10197 | ↑ |
| RBR1 | AT3G12280 | Bra034764 | 1,16114 | ↑ |
| RBR1 | AT3G12280 | Bra038707 | 1,13118 | ↑ |
| CDC20 | AT4G33270 | Bra011430 | 1,34962 | ↑ |
| CDC20 | AT4G33270 | Bra037011 | 1,09561 | ↑ |
| APC1 | AT5G05560 | Bra028755 | 1,36135 | ↑ |
| APC1 | AT5G05560 | Bra005857 | 1,48265 | ↑ |
| APC2 | AT2G04660 | Bra025616 | 1,55548 | ↑ |
| APC2 | AT2G04660 | Bra028905 | 1,75295 | ↑ |
| APC6 | AT1G78770 | Bra035062 | 1,13089 | ↑ |

**Supplementary Table 4.** Cell cycle genes (list from Bothwell et al. 2010) differentially expressed in *Maullinia ectocarpii* infected *Ectocarpus siliculosus* (Ec32m) in comparison to noninfected *Ectocarpus siliculosus*. The log two-fold changes (log2FC) are colour coded, with green as the highest value and red the lowest value. The upregulated genes are indicated with an upward arrow (↑) whilst the downregulated genes are highlighted with a downward arrow (↓). Significantly differentially expressed transcripts (p.adjusted values < 0.05 calculated by false discovery rate with Benjamini-Hochberg correction for multiple testing using DEseq2 v3.8) are indicated with a YES. Genes thought to be important for the switch from the mitotic cell cycle to the endocycle are highlighted in blue.

| Gene | Query | Hit/Gene ID | log2FC | significant | up/down |
| --- | --- | --- | --- | --- | --- |
| Ectsi FZR1 (CDH1-Ccs52) | esi0012_0096 | Ec-15_000510 | 0.04 | NO | ↑ |
| Ectsi Wee1 | esi0495_0011 | Ec-00_006420 | -1.42 | YES | ↓ |
| Ectsi CDKA1 | esi0037_0007 | Ec-04_005630 | 0.81 | YES | ↑ |
| Ectsi CDKA2 (CDKB) | esi0041_0093 | Ec-02_003750 | -0.26 | NO | ↓ |
| Ectsi CDKB ⁄ 4-like | esi0129_0022 | Ec-05_004440 | 0.20 | NO | ↑ |
| Ectsi CDKC1 | esi0007_0143 | Ec-08_002250 | 0.49 | YES | ↑ |
| Ectsi CDKC2,1 | esi0073_0098 | Ec-10_001350 | -0.30 | YES | ↓ |
| Ectsi CDKC2,2 | esi0010_0208 | Ec-20_004690 | 0.79 | YES | ↑ |
| Ectsi CDKD1 | esi0236_0041 | Ec-04_000570 | 0.59 | YES | ↑ |
| Ectsi CDKI1 | esi0191_0048 | Ec-13_000360 | -0.22 | NO | ↓ |
| Ectsi CDKH1 | esi0011_0074 | Ec-03_002400 | 0.53 | YES | ↑ |
| Ectsi CDK-related | esi0011_0004 | Ec-03_002760 | 0.34 | NO | ↑ |
| Ectsi CKS1 | esi0085_0076 | Ec-06_000360 | -0.37 | NO | ↓ |
| Ectsi CKS2 | esi0401_0014 | Ec-06_003970 | -2.10 | YES | ↓ |
| Ectsi CYCA1 | esi0228_0024 | Ec-01_000740 | -1.09 | YES | ↓ |
| Ectsi CYCB1 | esi0071_0052 | Ec-11_004920 | -1.86 | YES | ↓ |
| Ectsi CYCB2 | esi0295_0026 | Ec-01_009390 | -1.64 | YES | ↓ |
| Ectsi CYCD1 | esi0148_0011 | Ec-07_005740 | -4.80 | YES | ↓ |
| Ectsi CYCD2 | esi0220_0004 | Ec-11_004300 | 0.36 | NO | ↑ |
| Ectsi CYCD3 | esi0070_0096 | Ec-12_004410 | -0.20 | NO | ↓ |
| Ectsi CYCF1 | esi0057_0093 | Ec-07_000220 | 0.71 | YES | ↑ |
| Ectsi CYC | esi0064_0091 | Ec-06_003040 | -0.07 | NO | ↓ |
| Ectsi CYCH | esi0069_0006 | Ec-12_000030 | 0.47 | NO | ↑ |
| Ectsi CYCL1 | esi0091_0056 | Ec-22_002580 | -0.92 | YES | ↓ |
| Ectsi CYCT1 | esi0037_0063 | Ec-04_005240 | 0.07 | NO | ↑ |
| Ectsi CYCT2,1 | esi0119_0017 | Ec-11_001180 | 0.54 | YES | ↑ |
| Ectsi CYCT2,2 | esi0290_0030 | Ec-09_001090 | NA | NO |  |
| Ectsi DP | esi0063_0071 | Ec-14_006710 | -0.06 | NO | ↓ |
| Ectsi E2F | esi0014_0069 | Ec-21_005690 | 0.53 | NO | ↑ |
| Ectsi DEL | esi0250_0028 | Ec-17_001960 | -0.25 | NO | ↓ |
| Ectsi RBR | esi0089_0003 | Ec-14_002750 | -1.18 | YES | ↓ |
| Ectsi CDC20 | esi0047_0070 | Ec-16_002010 | -0.81 | NO | ↓ |
| Ectsi APC1 | esi0182_0021 | Ec-14_001510 | -0.32 | NO | ↓ |
| Ectsi APC2 | esi0044_0151 | Ec-04_003800 | |  |  |
| Ectsi APC3 | esi0331_0027 | Ec-03_001570 | 0.04 | NO | ↑ |
| Ectsi APC4 | esi0347_0010 | Ec-26_002710 | 0.27 | NO | ↑ |
| Ectsi APC5 | esi0255_0019 | Ec-06_009310 | 0.22 | NO | ↑ |
| Ectsi APC6 | esi0043_0021 | Ec-05_003300 | -0.42 | NO | ↓ |
| Ectsi APC7 | esi0203_0024 | Ec-01_008970 | 0.23 | NO | ↑ |
| Ectsi APC8 | esi0160_0056 | Ec-03_005170 | 0.06 | NO | ↑ |
| Ectsi APC10 | esi0035_0106 | Ec-19_002640 | 0.85 | YES | ↑ |
| Ectsi APC11 | esi0327_0018 | Ec-07_003050 | |  |  |

**Supplementary Table 5.** *Maullinia ectocarpii* genes related to the cell cycle (cog cat = D) which are predicted as effectors (EffectorP).

| **Gene** | **Gene expression** | **EffectorP** | **eggNOG** |  |  | **uniprot** |  | **blastp** |
| --- | --- | --- | --- | --- | --- | --- | --- | --- |
| ID | TPM | Prediction | COG cat | predicted_gene | annotation | protein | domain | % identity |
| TRINITY_DN12131_c1_g1_i1 | 608.51 | Effector | D | ANAPC10 | complex subunit 10 | Anaphase-promoting complex subunit 10 | DOC | 67.4 |
| TRINITY_DN32460_c0_g1_i1 | 217.38 | Effector | D | MOB1B | MOB kinase activator | Mps one binder kinase activator-like 1 protein | | 65.6 |
| TRINITY_DN28765_c0_g2_i1 | 83.14 | Effector | D | MOB1 | MOB kinase activator | Uncharacterized protein | | 76.6 |
| TRINITY_DN34562_c0_g1_i1 | 50.15 | Effector | D | NA | MOB kinase activator | uncharacterized Protein | | 57.5 |
| TRINITY_DN36819_c3_g2_i1 | 8.46 | Effector | D | NBP35 | Component of the cytosolic iron-sulfur (Fe S) protein assembly (CIA) machinery. Required for maturation of extramitochondrial Fe-S proteins. The NBP35-CFD1 heterotetramer forms a Fe-S scaffold complex, mediating the de novo assembly of an Fe-S cluster and its transfer to target apoproteins | Cytosolic Fe-S cluster assembly factor NUBP1 homolog | | 66.8 |
| TRINITY_DN28765_c0_g1_i1 | 4.27 | Effector | D | MOB1 | MOB kinase activator | Uncharacterized protein | | 84.0 |
| TRINITY_DN31047_c0_g1_i1 | 2.29 | Effector | D | CHEK2 | serine threonine-protein kinase | Protein kinase domain-containing protein | Protein kinase | 51.9 |
| TRINITY_DN26027_c0_g2_i1 | 1.44 | Effector | D | DCLK3 | doublecortin-like kinase | Protein kinase domain-containing protein | Protein kinase | 72.3 |
| TRINITY_DN28783_c0_g4_i1 | 0.22 | Effector | D | NA | Cyclin K | Uncharacterized protein | Cyclin | 51.6 |

**Supplementary Table 6.** Cell cycle related genes in *Plasmodiophora brassicae* which act as potential effectors (predicted by EffectorP) (on their host *Brassica oleracea*).

| **Gene** | **Gene expression** | **EffectorP** | **eggNOG** | | | **kegg** | | **blastp** |
| --- | --- | --- | --- | --- | --- | --- | --- | --- |
| ID | FPKM_YG | Prediction | COG cat | predicted_gene_name | eggNOG annot | KEGG_identifier | KEGG_description | % identy |
| TRINITY_DN122143_c1_g1_i1 | 71.92 | Effector | D | NA | Mitotic checkpoint protein | BUB3 | cell cycle arrest protein BUB3 | 100 |
| TRINITY_DN34123_c0_g1_i1 | 60.08 | Effector | D | AURKA | serine threonine-protein kinase | NA | NA | 98.266 |
| TRINITY_DN34569_c1_g1_i1 | 37.74 | Effector | D | CDC25B | cell division cycle 25 homolog | MIH1 | M-phase inducer tyrosine phosphatase [EC:3.1.3.48] | 100 |
| TRINITY_DN115607_c1_g1_i1 | 26.16 | Effector | D | BRUCE | Baculoviral IAP repeat containing | BIRC6, BRUCE | baculoviral IAP repeat-containing protein 6 (apollon) [EC:2.3.2.23] | 100 |
| TRINITY_DN54734_c0_g1_i1 | 25.52 | Effector | D | NA | MOB family member 4, phocein | NA | NA | 100 |
| TRINITY_DN41093_c0_g2_i1 | 21.31 | Effector | D | APC7 | anaphase promoting complex subunit 7 | APC7 | anaphase-promoting complex subunit 7 | 99.587 |
| TRINITY_DN80052_c0_g2_i1 | 11.34 | Effector | D | TTK | ttk protein kinase | TTK, MPS1 | serine/threonine-protein kinase TTK/MPS1 [EC:2.7.12.1] | 100 |
| TRINITY_DN120696_c1_g1_i1 | 7.39 | Effector | D | WDR74 | WD repeat domain 74 | NA | NA | 100 |
| TRINITY_DN77936_c1_g1_i1 | 7.15 | Effector | D | NA | cysteine | NA | NA | 100 |
| TRINITY_DN11234_c0_g1_i1 | 6.64 | Effector | D | RPTOR | regulatory associated protein of MTOR | RAPTOR | regulatory associated protein of mTOR | 98.745 |
| TRINITY_DN86908_c0_g2_i2 | 5.37 | Effector | D | SMC1 | structural maintenance of chromosomes protein | SMC1 | structural maintenance of chromosome 1 | 100 |
| TRINITY_DN58085_c0_g2_i1 | 5.34 | Effector | D | ANAPC10 | complex subunit 10 | APC10, DOC1 | anaphase-promoting complex subunit 10 | 100 |

**Supplementary Table 7.** FISH probes used in the experiment, including the target, the sequence, the stain and the excitation wavelength.

| probe | target | sequence | stain | excitation wavelength |
| --- | --- | --- | --- | --- |
| Pl_LSU_2313 | 28S rDNA of *P. brassicae* | 5’- CCAGGCCTTTCAGCCAAGTA -3‘ | 6-FAM | 490 nm |
| MauJ17 | 18S rDNA of *M. ectocarpii* | 5’- CACGTCCCTCGTACCCGT -3’ | 6-FAM | 490 nm |

**Supplementary Results**

**Colonization of cells by *P. brassicae* induces endocycle related transcriptional changes in *Brassica* hosts**

By querying available RNA-seq datasets we could find genetic signatures pointing towards the induction of endocycle related processes in *Brassica oleracea subsp. gongylodes* and *B. rapa subsp. pekinensis* infected by *P. brassicae* (data from Ciaghi et al., 2019; Jia et al., 2017). In those datasets the transcripts linked to the switch from the mitotic cell cycle to the endocycle (as described for *A. thaliana* by Olszak et al., 2019) were generally upregulated. CCS52A1 and CCS52B, activators of the anaphase-promoting complex/cyclosome (APC/C) and involved in the switch from mitotic cycle to the endocycle, were upregulated in infected *B. oleracea subsp. gongylodes* plants, as was CCS52A1 in infected *B. rapa subsp. pekinensis,* confirming the observations in *P. brassicae* infected *A. thaliana* plants from Olszak et al. 2019 (Table 1). CDC20 another activator of the APC/C was upregulated in both infected *Brassica* species as well and, as a consequence, transcripts of the APC/C were upregulated (Supplementary Table 1, Supplementary Table 2). The protein kinase WEE1, which is involved (among other biological processes) in a different pathway leading to a switch from the mitotic cell cycle to the endocycle, was also upregulated in infected *Brassicas* (Table 1, Supplementary Table 1, Supplementary Table 2). Transcripts of genes for G1 to S progression (i.e., mainly involved in the replication of the DNA) were upregulated in infected plants compared with uninfected ones (Supplementary Table 1, Supplementary Table 2). Transcripts of cyclin D, which are expressed during the G1 and S phase, were upregulated in *B. oleracea subsp. gongylodes* and *B. rapa subsp. pekinensis* (both up and down regulated in infected *A. thaliana* (Olszak et al. 2019)) (Supplementary Table 1, Supplementary Table 2). E2Fa, a transcriptional activator of G1/S specific genes, was upregulated in infected plants (Table 1). DPa, its dimerization partner, was also upregulated in infected *Brassicas* (Supplementary Table 1, Supplementary Table 2). CDKA important for both the G1/S phase transition and the G2/M phase transition, was upregulated in infected *B. oleracea subsp. gongylodes* plants (Table 1). Transcripts associated to G2/M specific genes important for the transition to the M-phase did not show a clear pattern of regulation, they were upregulated in infected *B. oleracea subsp. gongylodes* and *B. rapa subsp. pekinensis*; and both up- and downregulated in infected *A. thaliana* (Olszak et al. 2019). G2/M specific CDKB transcripts and cyclins (CYCBs, some CYCAs) were upregulated (Table 1).

**Supplementary Methods**

**Flow cytometry**

Flow cytometry was performed as described in (Suda et al., 2007) with some modifications explained in the supplementary methods. The used standards (*Bellis perennis* for *Brassica rapa subsp. pekinensis* and *Solanum pseudocapsicum* for *Ectocarpus siliculosus*) were used because of their similar but not overlapping genome size with the used material

Plants (*Plasmodiophora brassicae* in *Brassica rapa subsp. pekinensis)*

Fresh root galls of *B. rapa subsp. pekinensis* infected with *P. brassicae* were rinsed in tap water. Roots of noninfected *B. rapa subsp. pekinensis* were used as a control and treated in the same way. Roots were chopped together with a standard (*Bellis perennis*) (Schönswetter et al., 2007) in ice cold Otto 1 buffer (0.1 M citric acid, 0.5% Tween 20). The nuclear suspension was filtered through a 42 µm nylon mesh and 1mL Otto2 buffer (0.4 M Na_2_HPO_4_ 12 H_2_O), supplemented with 4’, 6 – diamidino – 2 – phenylindole (DAPI) (Sigma, USA, 4 µg/mL) and 2-mercaptoethanol (2 µl/mL), was added. The relative fluorescence intensity of 3000 particles was measured with a CyFlow space flow cytometer (Sysmex Partec GmbH, Germany) equipped with a UV LED 365 nm. Histograms were analyzed using the FloMax software (Partec GmbH, Germany).

Algae (*Maullinia ectocarpii* in *Ectocarpus siliculosus*)

*E. siliculosus* Ec32m infected with *M. ectocarpii* was used for flow cytometric measurements. Uninfected *E. siliculosus* Ec32m cultures were used as a control. Fresh algal material was chopped together with the standard *Solanum pseudocapsicum* in modified NIB/2 buffer (NIB: pH 7.5, Sorbitol 125 mM, Potassium Citrate 20 mM, Magnesium Chloride 30 Mm, Hepes 55Mm, EDTA 5mM) supplemented with TritonX- 100 (0.1%) and PVP (1%). The nuclear suspension was filtered through a 42 µm nylon mesh and 1mL modified NIB/2 buffer supplemented with TritonX- 100 (0.1%), PVP (1%) and DAPI (Sigma, USA, 4 µg/mL) was added. The relative fluorescence intensity of 3000 particles was measured with a CyFlow space flow cytometer (Sysmex Partec GmbH, Germany) equipped with a UV LED 365 nm. Histograms were analyzed using the FloMax software (Partec GmbH, Germany).

**Identification of cell cycle related genes in infected hosts**

Three publicly available RNA-seq datasets were analyzed to examine the cell cycle-related genes in phytomyxid-infected hosts. The first was from *Brassica oleracea subsp. gongylodes* infected by *Plasmodiophora brassicae* ((Ciaghi et al., 2019); BioProject: PRJEB26435), the second from *Brassica rapa subsp. pekinensis* ((Jia et al., 2017); Bioproject: PRJNA322393), and the third from *Ectocarpus siliculosus* Ec32m (strain CCAP 1310/4) infected by *Maullinia ectocarpii* (strain CCAP 1538/1; (Garvetto et al., 2023); BioProject: PRJNA878940). The two transcriptomes (and inferred proteomes) from *B. oleracea subsp. gongylodes* and *E. siliculosus* Ec32m were transformed into blast databases and queried via BLAST (Altschul et al., 1990) for genes (*blastn*) and proteins (*blastp*) homologous to sequences of cell cycle-related genes involved in endoreduplication described by (Olszak et al., 2019) and (Bothwell et al., 2010). Hits were filtered using identity threshold with an identity higher than 50 % for peptides and equal or higher 80% for transcripts and their peptide sequences were blasted against the NCBI database and UniProt database. Resulting gene lists for *B. oleracea* and *E. siliculosus* were compiled and reciprocal keyword searches based on gene models (from *E. siliculosus* to *B. oleracaea* and vice versa) were used to identify additional potential cell cycle-related homologous. For *B. rapa subsp. pekinensis*, the complete list of genes differentially expressed in the interaction between susceptible plants and *P. brassicae* (supplementary table S2 in (Jia et al., 2017)) were queried using *B. rapa* homologues of the reference gene models used for *B. oleracea subsp. gongylodes* (Supplementary Table 3). Additionally, log2fold change values were extracted (Supplementary Table 2, Supplementary Table 3, Supplementary Table 4) and analyzed to investigate the behavior of cell cycle-related genes in Phytomyxea-infected hosts. The most important genes involved in endoreduplication were summarized in **Supplementary Table 1** and compared with literature. Potential effectors were identified by filtering the datasets (*M. ectocarpii* and *P. brassicae*) via the cog category D (for cell cycle) together with the EffectorP prediction (only hits from the category effector were kept) Additionally only hits with a peptide identity higher than 50% were kept (Supplementary Table4, Supplementary Table5
